# A New Perceptual Bias Reveals Suboptimal Population Decoding of Sensory Responses

**DOI:** 10.1371/journal.pcbi.1002453

**Published:** 2012-04-12

**Authors:** Tom Putzeys, Matthias Bethge, Felix Wichmann, Johan Wagemans, Robbe Goris

**Affiliations:** 1 Laboratory of Experimental Psychology, University of Leuven, Leuven, Belgium; 2 Werner Reichardt Centre for Integrative Neuroscience and Institute of Theoretical Physics, University of Tübingen, Tübingen, Germany; 3 Max Planck Institute for Biological Cybernetics, Computational Vision and Neuroscience Group, Tübingen, Germany; 4 Bernstein Centre for Computational Neuroscience, Tübingen, Germany; 5 AG Neuronale Informationsverarbeitung, Mathematisch-Naturwissenschaftliche Fakultät, Eberhard Karls Universität Tübingen, Tübingen, Germany; 6 Max Planck Institute for Intelligent Systems, Abteilung Empirische Inferenz, Tübingen, Germany; 7 Center for Neural Science, New York University, New York, New York, United States of America; Northwestern University, United States of America

## Abstract

Several studies have reported optimal population decoding of sensory responses in two-alternative visual discrimination tasks. Such decoding involves integrating noisy neural responses into a more reliable representation of the likelihood that the stimuli under consideration evoked the observed responses. Importantly, an ideal observer must be able to evaluate likelihood with high precision and only consider the likelihood of the two relevant stimuli involved in the discrimination task. We report a new perceptual bias suggesting that observers read out the likelihood representation with remarkably low precision when discriminating grating spatial frequencies. Using spectrally filtered noise, we induced an asymmetry in the likelihood function of spatial frequency. This manipulation mainly affects the likelihood of spatial frequencies that are irrelevant to the task at hand. Nevertheless, we find a significant shift in perceived grating frequency, indicating that observers evaluate likelihoods of a broad range of irrelevant frequencies and discard prior knowledge of stimulus alternatives when performing two-alternative discrimination.

## Author Summary

An attractive view on human information processing proposes that inference problems are dealt with in a statistically optimal fashion. This hypothesis can explain aspects of perception, movement planning, cognition and decision making. In the present study, I use a new psychophysical paradigm that reveals surprisingly suboptimal perceptual decision making. Observers discriminate between two sinusoidal gratings of a different spatial frequency. Making use of visual noise, I induce an asymmetry in neural population responses to the gratings and find this asymmetry to effectively bias perceptual decision making. A simple ideal observer model, uninformed about the presence of visual noise but only considering the two grating spatial frequencies relevant to the task at hand, manages to avoid such a bias. I conclude that observers are limited in their ability to make use of prior knowledge of relevant visual features when performing this task. These results are in line with a growing number of findings suggesting that near-optimal decoders, although straightforward to implement and achieving near-maximal performance, consistently overestimate empirical performance in simple perceptual tasks.

## Introduction

Perceptual decisions in a wide range of visual tasks ultimately rely on information encoded in neural responses in primary visual cortex (V1). However, this information may not be readily available to higher levels of the visual system because it is distributed across entire populations of neurons. Moreover, each neuron's reliability is limited by intrinsic response variability and its relevance strongly depends on the perceptual task in which the organism is engaged. To form accurate perceptual judgements, the brain thus needs to pool sensory responses efficiently, decoding the population response into a reliable decision variable.

A wide range of psychophysical and physiological studies have investigated population decoding efficiency in simple visual tasks such as two-alternative detection and discrimination [1]. Results are not unambiguous: some studies suggest that the visual system uses a flexible precision pooling scheme in which the contribution of sensory responses to perceptual decisions depends on their reliability and relevance to the task at hand [2]–[7]. However, other studies [8]–[11] do not report such optimal decoding but rather suggest a crude, unselective pooling scheme in which the decision pool includes sensitive as well as many insensitive neurons. It is not clear why results differ across studies, which illustrates that neural decision making is, as yet, not fully understood.

A crucial issue concerns the neural implementation of optimal population decoding. Formally, optimal decoding requires to evaluate neural responses probabilistically by computing the (log) likelihood function. This function captures the likelihood that specific stimuli gave rise to the observed population response. Theoretical work has shown that, under certain conditions, the necessary part of the log likelihood function can be obtained through simple linear combination of neural responses [12], [13]. However, computing the likelihood function only solves part of the decoding problem. Subsequently, the likelihood function has to be read out and linked to a decision variable [1]. In two-alternative discrimination tasks, only two stimulus alternatives need to be considered. An optimal decoder aiming to maximize accuracy therefore calculates a ratio of likelihoods by reading out the likelihood function precisely at two specific locations that correspond to the two possible stimulus alternatives. Likewise, in a detection task in which the quantity of the stimulus to be detected is known, such a decoder only considers the likelihood of the relevant stimulus quantity being present or absent. Formally, retrieving the likelihoods of two discrete stimulus values corresponds to integrating the likelihood function with appropriately placed and infinitely narrow read-out functions. While the use of a likelihood ratio decision variable has been demonstrated in various perceptual tasks [12], [14]–[16], it remains unclear to what extent the likelihood ratio can be computed with arbitrary high precision. A failure to sample the likelihood representation with high precision may account for the suboptimal unselective pooling reported previously.

Here, we estimate the width of the likelihood read-out functions involved in two-alternative pattern detection and discrimination by modeling a new perceptual bias. We measured grating detectability and discriminability in the presence of filtered visual noise and found that filtered noise backgrounds dramatically alter the perception of grating spatial frequency. Embedding a grating in low-pass filtered noise causes the perceived spatial frequency of the grating to decrease, while high-pass filtered noise increases perceived grating spatial frequency. A population code model is proposed, consisting of a physiologically inspired V1 encoding front-end followed by a decoding stage. By simultaneously modeling grating detectability and discriminability, we were able to estimate both the amount of sensory information encoded in the V1 population response as well as the extent to which this information is used to maximize performance. While filtered visual noise induces an asymmetry in the likelihood function, an ideal observer only evaluates the likelihood of exactly the two possible stimuli and is thus not affected by this asymmetry. Therefore, the observed bias indicates severely suboptimal decoding which we assign to an imprecise read-out of the likelihood function. Only when read-out functions as broad as 4 octaves are assumed, the model accounts for all behavioural measurements. These findings suggest that observers failed to sample the likelihood function at the appropriate locations with arbitrary high precision and consequently, that precision pooling was not adopted in our tasks.

## Results

### Spatial frequency perception is biased in filtered visual noise

**Figure 1:**
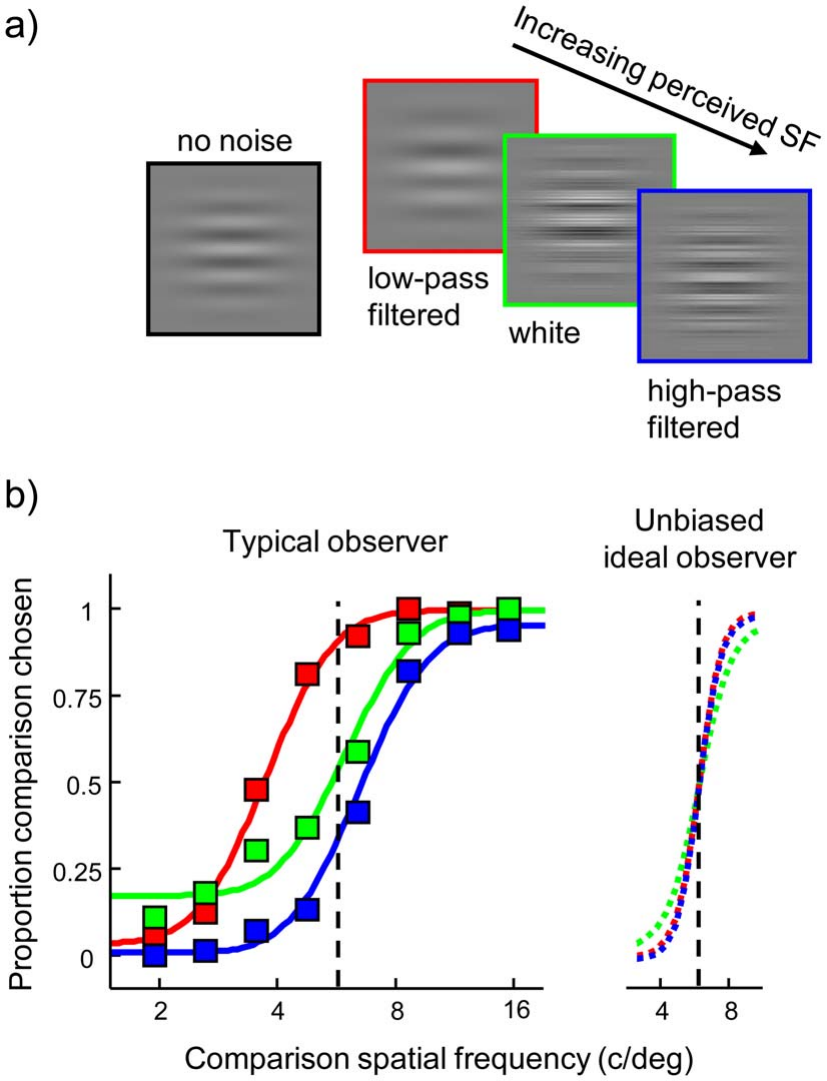
Spatial frequency perception is biased in filtered noise. (a) The perceived spatial frequency of a grating embedded in low-pass noise (red) and high-pass noise (blue) is respectively lower and higher than the perceived spatial frequency of the same grating embedded in white noise (green). (b) Left panel: results of a typical observer (BM). The spatial frequency of a comparison grating embedded in white noise was varied to match a standard grating of 5.5 c/deg in low-pass filtered, high-pass filtered and white noise. In low-pass and high-pass filtered noise, a respectively lower and higher comparison frequency is required to match the standard grating, indicating that perceived standard grating frequency is biased in the direction of the noise. Full lines represent the best-fitting Weibull psychometric functions. Right panel: an ideal observer model adopting optimal narrow read-out functions to sample the likelihood function predicts unbiased discrimination performance.

We measured the effect of filtered noise on perceived spatial frequency in a two-alternative two-interval discrimination task. One grating was embedded in low-pass or high-pass filtered 1-D noise (cut-off at 5.5 cycles per degree) while another grating was presented in unfiltered, broadband noise (Figure 1a). By varying the spatial frequency of one grating (the comparison grating) while keeping the other grating (the standard grating) constant, we obtained the relative matching frequency. This measure indicates the perceived spatial frequency of a grating in filtered noise relative to a grating in broadband noise. Conditions in which the standard grating was embedded in filtered noise while the comparison grating was presented in broadband noise and vice versa were randomly intermixed (see methods).

**Figure 2:**
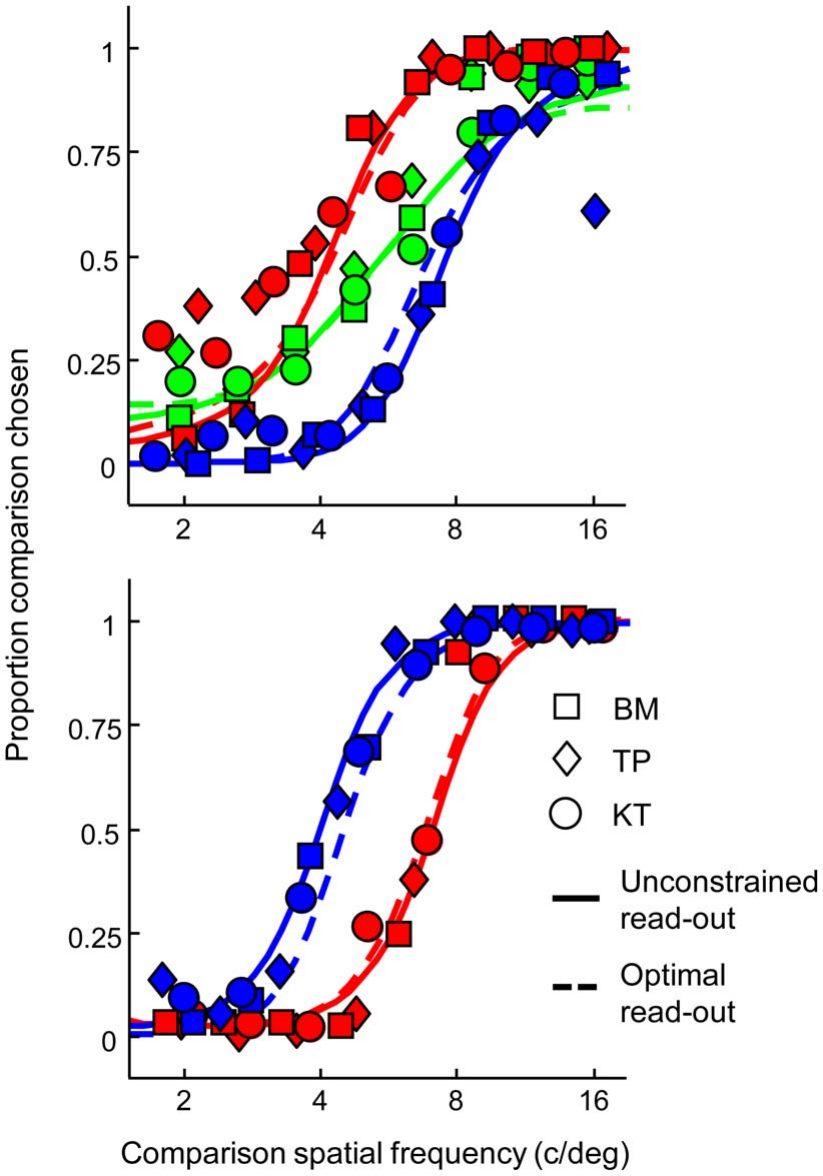
Spatial frequency discrimination data and model fits. Symbols indicate the pooled data of individual observers obtained in the main (top panel) and control (bottom panel) conditions of the discrimination experiment. Red, green and blue colors refer respectively to the low-pass filtered, white and high-pass filtered noise conditions. Full lines represent the fit of our population code model in which likelihood read-out function width is a free parameter. Broken lines denote the fit when narrow read-out functions are assumed in the same model. Our model accounts for the perceptual bias by assuming that the log likelihood function is read out with limited precision (full lines). When high-precision read-out is implemented, the model manages to capture the bias to a reasonable extent (broken lines) by severely reducing the amount of encoded spatial frequency information. It should be noted that the model fails to account for grating detectability in the latter case (see also Figure 3).

In the former conditions (hereafter referred to as the main conditions), low-pass noise significantly decreased perceived standard grating spatial frequency (parametric Monte-Carlo test, 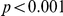 for all observers, see methods) from 5.5 cycles per degree (c/deg) to an average of 3.8 c/deg (s.e.m. = 0.63). Conversely, high-pass filtered noise increased perceived spatial frequency (parametric Monte-Carlo test, 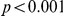 for all observers) to an average of 7.2 c/deg (s.e.m. = 0.64). The average bias across observers equalled −0.5 octaves (s.e.m. = 0.07) in the low-pass filtered noise condition and 0.4 octaves (s.e.m. = 0.08) in the high-pass noise condition. The data of a typical subject are shown in Figure 1b. As all subjects displayed a similar perceptual bias, data were pooled across observers to increase statistical power (see supporting text S2). The pooled data, displayed in Figure 2, will be used in the remainder of this study. A demonstration of the bias is provided in Figure 1a.

In the conditions in which the comparison grating instead of the standard grating was embedded in filtered noise, a similar bias was found (Figure 2, bottom panel). Embedding the comparison grating in low-pass filtered noise decreased its perceived spatial frequency (parametric Monte-Carlo test, 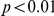 for all observers), requiring an average comparison frequency of 6.6 c/deg (s.e.m. = 0.61) to match the 5.5 c/deg standard grating embedded in broadband noise. Perceived grating frequency increased in high-pass filtered noise (parametric Monte-Carlo test, 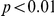 for all observers), resulting in a matching frequency of 4.2 c/deg (s.e.m. = 0.62). The average bias due to low-pass and high-pass filtered noise in the latter conditions equalled respectively −0.4 (s.e.m. = 0.05) and 0.4 (s.e.m. = 0.05) octaves. Overall performance was significantly higher in the control conditions compared to the main conditions (86% vs. 78% correct, binomial test, 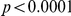). Correspondingly, a smaller bias and steeper psychometric functions were observed in the control conditions for all observers, but these differences were not statistically significant.

**Figure 3:**
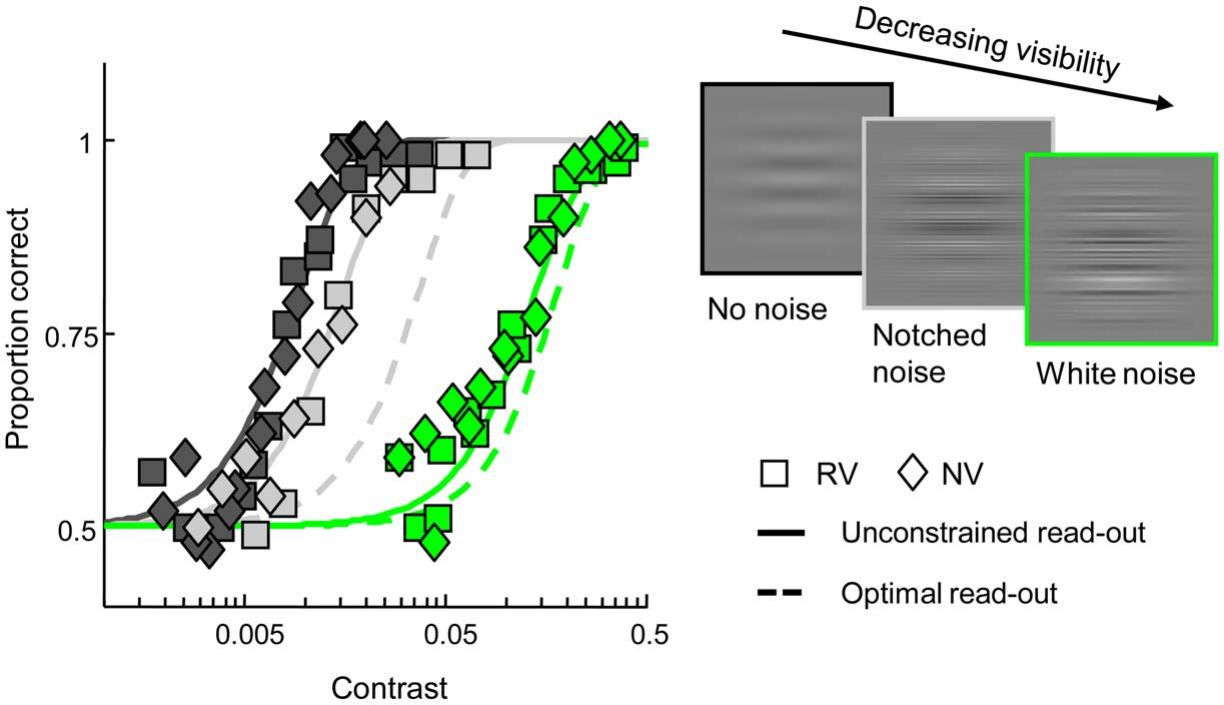
Contrast detection data and model fits. White noise (green) impairs detection performance considerably, while detectability in notched noise (gray) and in the absence of external noise (black) are approximately equal. Symbols indicate the pooled data of individual observers. Full lines represent the fit of our population code model in which likelihood read-out function width is a free parameter. Broken lines denote the fit when narrow read-out functions are assumed in the same model. Our population code model captures contrast detection performance in all conditions when low-precision likelihood read-out is assumed (full lines). The same model implementing high-precision read-out underestimates grating detectability in notched noise and white noise (broken lines).

In addition, we measured the visibility of the 5.5 c/deg standard grating under various noise conditions in a two-alternative two-interval contrast detection task (see methods). In the absence of visual noise, the contrast required to achieve 75% correct detection performance equalled 0.8% on average across subjects (s.e.m. = 0.04%). Embedding the grating in the broadband noise backgrounds used in our discrimination task increases the detection threshold contrast to an average of 10.3% (s.e.m. = 0.005%). We also determined grating visibility in the presence of filtered notched noise, i.e., broadband noise from which a 4-octave-wide notch centred around 5.5 c/deg has been removed. The average detection threshold in the latter condition equalled 1.3% (s.e.m. = 0.26%). The pooled detection data are provided in Figure 3.

### A population code model for spatial frequency perception

**Figure 4:**
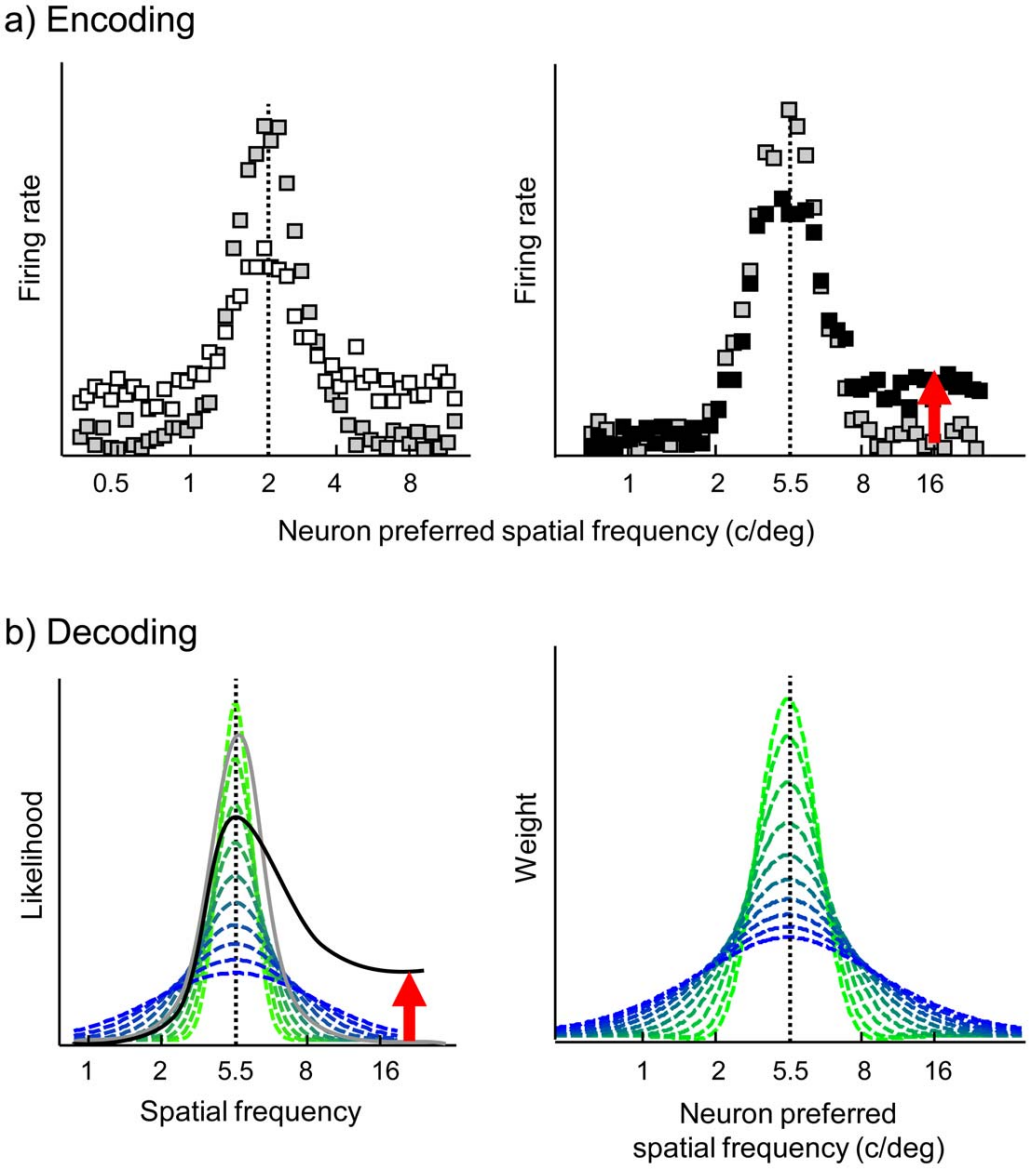
Overview of the population code model. (a) Model of V1 encoding of gratings in filtered noise. Left panel: simulated V1 responses to a grating of 2 c/deg in the absence of external noise (gray symbols) and in the presence of white noise (white symbols). Right panel: simulated V1 responses to a grating of 5.5 c/deg in the absence of noise (gray symbols) and in the presence of high-pass filtered noise (black symbols). It can be seen that visual noise reduces the response of informative neurons tuned to the grating frequencies while increasing the activity of neurons tuned to the noise pass-band. Consequently, the population response is asymmetrical in filtered noise. (b) Decoding of grating spatial frequency. Left panel: Reduced log likelihood function for the population response to a 5.5 c/deg grating in the absence of external noise (gray line) and in the presence of high-pass filtered noise (black line). Embedding the grating in high-pass noise increases the log likelihood of high spatial frequencies relative to the veridical frequency of 5.5 c/deg. The reduced log likelihood function is thus asymmetrical in filtered noise. The broken lines represent the read-out functions assumed when sampling discrete likelihood values. Right panel: the linear weighting profiles corresponding to the read-out functions. The likelihood of a specific grating spatial frequency can be obtained with high precision (left panel, green) by preferentially weighting the responses of neurons that are tuned to that specific frequency (right panel, green). Computing the likelihood of a grating spatial frequency with low precision (left panel, blue) corresponds to summing the responses of a broad population of neurons tuned to various spatial frequencies (right panel, blue).

Detection and discrimination of gratings presumably depend on the responses of V1 neurons, which are relatively sharply tuned to spatial frequency [17]–[19]. We implemented a descriptive model consistent with the normalization model of simple cells [20] as an encoding front-end to simulate the responses of V1 neurons to gratings embedded in filtered visual noise (see methods). Our model provides a description of the contrast response of typical V1 neurons, incorporating Gabor-like linear excitatory receptive fields tuned to spatial frequency [21], nonlinear response transduction [22] and the inhibitory effects of broadly-tuned contrast gain control [23]. The model captures the effects of broadband visual noise on the contrast response function [20], [24]–[29]. The main effect is a substantial shift towards higher contrasts and lower response rates, corresponding to inhibition of informative neural responses. Broadband noise additionally induces a mild elevation of the spontaneous discharge in neurons tuned to the noise, thereby increasing the amount of uninformative neural responses. These effects are predicted by our model, in which strong inhibition is due to the activation of the broadly-tuned contrast gain control mechanism. Conversely, the mild elevation of spontaneous discharge results from activation of relatively sharply-tuned excitatory receptive fields. Extending the logic of the normalization model to filtered noise, our model predicts either excitation or inhibition depending on whether the noise stimulates the neurons' excitatory receptive field or gain control mechanism. For instance, the population responses simulated by the model are asymmetric in low-pass and high-pass filtered noise (Figure 4a) as these noise backgrounds selectively increase the activation of neurons tuned to frequencies respectively below and above 5.5 c/deg. In the presence of notched noise backgrounds, the model predicts excitation of neurons tuned to spatial frequencies below 1.4 c/deg or above 22 c/deg. Neurons tuned to the inside of the notch, on the other hand, are inhibited as the noise mainly activates the broadly-tuned gain control mechanism. Most aspects of the encoding front-end, i.e., spatial frequency tuning bandwidth of the cells' excitatory receptive field and the inhibitory gain control pool, the cells' contrast threshold as well as the absolute amount of noise excitation and inhibition, are controlled by free parameters.

In a decoding stage, simulated population responses were recoded into a log likelihood function of spatial frequency (see methods and supporting text S1). Decisions in both the detection and discrimination task are assumed to depend on a likelihood ratio decision variable, which is obtained by reading out the likelihood function at specific grating spatial frequencies using Gaussian-shaped read-out functions. These functions are assumed to be centred at the correct grating spatial frequencies and their width was included as a free parameter in the model. An optimal decoder implements infinitely narrow read-out functions and hence only considers the likelihood of relevant grating frequencies that actually can occur within a given block of trials. A decoder implementing broad read-out functions evaluates likelihood averaged over a broad range of grating frequencies that have zero prior probability. It should be noted that equal read-out function widths were used to model detection and discrimination unless stated otherwise.

Neural and computational constraints may prevent the visual system from computing the likelihood function optimally. We therefore consider a simplified biologically plausible likelihood computation [12]. When computing log likelihood, the decoder assumes Poisson noise and is ignorant about the effect of the filtered noise on the population response (see supporting text S1.2.4). A major advantage of these simplifications is that the part of the log likelihood function relevant to the decision variable in our tasks reduces to a linear function of the population response (see supporting text S1.2.2). We hereafter refer to this part as the reduced log likelihood function. This function can be obtained through precision pooling, only requiring knowledge of neuron spatial frequency tuning functions. More specifically, the reduced log likelihood of a specific spatial frequency equals the sum of the responses of all neurons tuned to that specific frequency. Neurons tuned to other frequencies provide no evidence for the presence or absence of the spatial frequency under consideration and are to be ignored. This is demonstrated in Figure 4: a strong response of neurons tuned to 5.5 c/deg (Figure 4a, right panel, gray symbols) provides strong evidence for the presentation of a 5.5 c/deg grating. The likelihood of 5.5 c/deg is large (Figure 4b, left panel, gray line) and can be obtained by summing the responses of neurons tuned to 5.5 c/deg. More specifically, the optimal weighting profile provided in Figure 4b (right panel, green) has to be applied to the population response in order to weight neurons according to their sensitivity to 5.5 c/deg. If spatial frequency tuning functions are assumed to be Gaussian-shaped and of equal bandwidth, the optimal weighting profile is also Gaussian-shaped, centred at the relevant spatial frequency and has a bandwidth that is proportional to the spatial frequency tuning bandwidth (see supporting text S1.2.2). It should be noted that the optimal weighting profile provided in Figure 4b (right panel, green) captures the neurons' sensitivity to exactly 5.5 c/deg. This weighting profile thus implies infinitely narrow likelihood read-out functions. Broader read-out functions (Figure 4b, left panel, blue) can be implemented by broadening the weighting profile as shown in Figure 4b (right panel, blue), resulting in lower-precision pooling (see supporting text S1.3).

As mentioned earlier, the decoder is ignorant about the presence of filtered noise. Low-pass and high-pass filtered noise backgrounds increase the response of neurons tuned to low and high spatial frequencies and hence increase the likelihood of low and high spatial frequencies. The reduced log likelihood function is thus asymmetrical in filtered noise. This is illustrated in Figure 4: neurons tuned to 16 c/deg increase the likelihood of a 16 c/deg grating (Figure 4b, left panel, black line), even when those neurons are actually responding to a high-pass filtered noise background added to a 5.5 c/deg grating (Figure 4a, right panel).

To isolate possible effects of variables such as correlated noise, pooling noise, late decision noise or general attention level, mainly affecting overall observer efficiency, we additionally included a late efficiency parameter to rescale the signal-to-noise ratio of the decoder by a constant factor.

### Evaluation of model fit

The best-fitting model accurately predicts the bias in perceived spatial frequency in the discrimination task (Figure 2, full lines) while simultaneously capturing grating visibility in the detection task (Figure 3, full lines). Akaike's Information Criterion (AIC) was used to evaluate the quality of the model fit while taking into account model complexity formalized as the amount of free parameters. To provide an upper limit on model performance given the variability inherent in the data, we computed AIC for the best-fitting theory-free and thus highly flexible Weibull psychometric function model. AIC of our model is not significantly higher than the AIC of the Weibull model (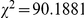, parametric Monte-Carlo test, 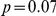), suggesting a relatively good model fit given the variability in the data and the limited number of free parameters.

### Physiologically-plausible encoding of spatial frequency information

**Figure 5:**
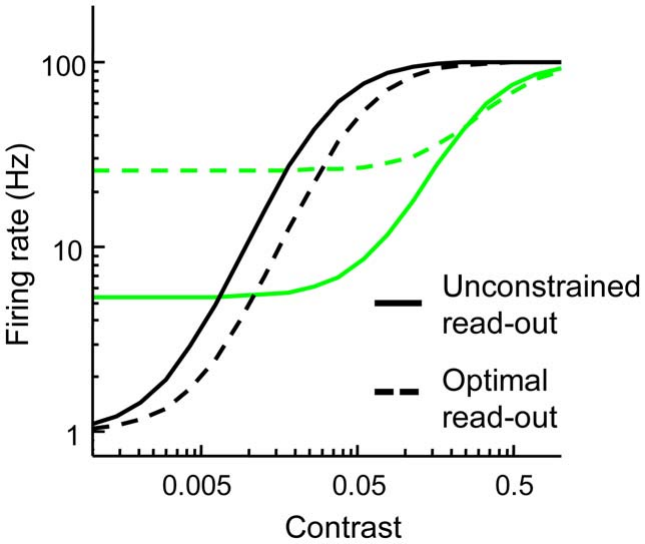
Best-fitting contrast response functions assumed in our population code model in which read-out function width is either a free parameter (full lines) or infinitely narrow (broken lines). Green and black lines respectively indicate the contrast response to gratings embedded in white noise and presented in the absence of noise.

Remarkably, the estimates of the encoding stage parameters lie well within the range of values reported in monkey or cat V1 single-cell recording studies. Spatial frequency tuning bandwidth of the excitatory receptive field was estimated at 0.9 octaves. DeValois, Albrecht and Thorell [21], for instance, report that a considerable portion of measured V1 cells had an excitatory receptive field bandwidth between 0.5 and 1.5 octaves. The estimated bandwidth of the inhibitory gain control mechanism is considerably broader, equalling 2.9 octaves. The fact that our gain control pool is not infinitely broad implies a degree of frequency-specific suppression, which has been suggested in multiple physiological studies [30]–[32]. The cells' semisaturation contrast is estimated at 3%, in agreement with estimates of human V1 semisaturation contrast [33]. Our model predicts a spontaneous discharge rate of 5.3 Hz in broadband noise and a shift of the contrast response function towards higher contrasts and lower response rates, leading to an average response reduction of approximately a factor of four at grating contrast levels between 10% and 30% (Figure 5). Touryan, Lau and Dan [28] measured an average spontaneous discharge of approximately 6 Hz to 1-D broadband noise stimuli in cat V1 and report a four-fold response reduction. Rust et al. [27] measured a three-fold response reduction in macaque V1 using similar 1-D noise stimuli.

### Suboptimal decoding involving broad read-out functions and low-precision pooling

**Figure 6:**
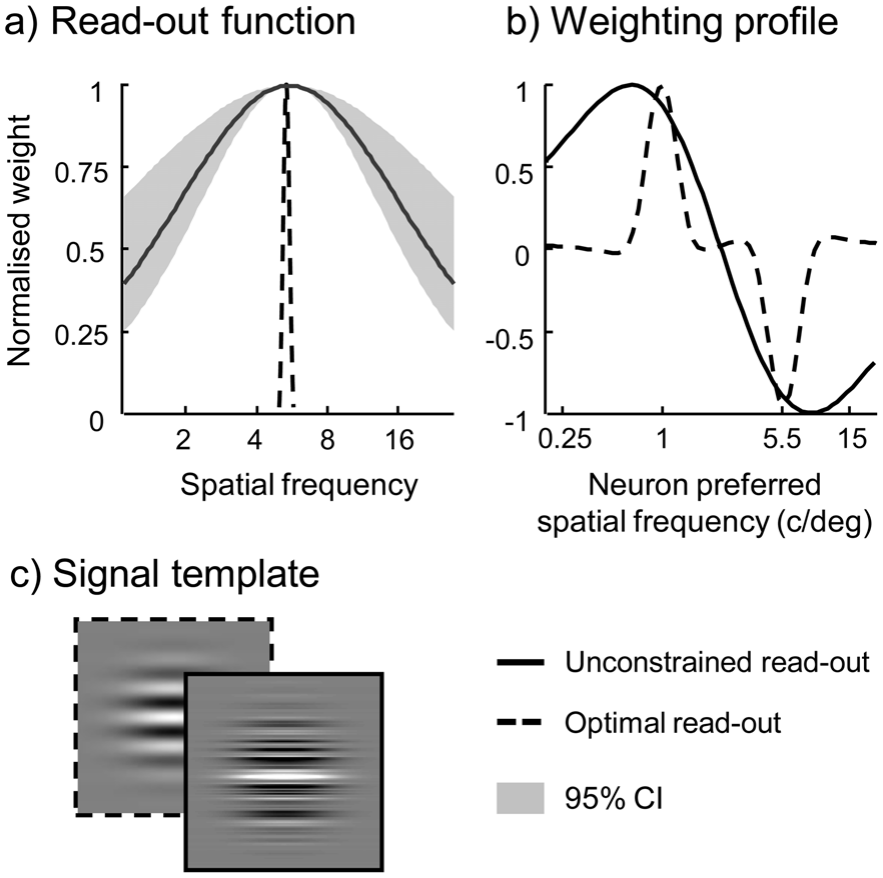
(a) Our population code model accounts for the perceptual bias and captures grating detectability in the presence and absence of external noise using a single set of parameters when low-precision likelihood read-out is assumed. The full line represents the best-fitting read-out function to detect or discriminate a 5.5 c/deg grating. The broken line denotes the optimal, infinitely narrow read-out function. (b) The linear weighting profile for discrimination between 1 c/deg and 5.5 c/deg that is consistent with the broad read-out function in (a). The dotted line represents the optimal weighting profile for the best-fitting model. (c) An example signal template consistent with the read-out function displayed in (a).

Crucially, the estimated read-out functions deviate considerably from optimal, infinitely narrow delta functions. Their width is estimated at 4.2 octaves (95% CI ranging from 3.4 to 4.8 octaves, Figure 6a), suggesting that the log likelihood function is read out with limited precision. Observers consider the log likelihood values of spatial frequencies that are a-priori unlikely to occur during the tasks, which lowers grating detectability and leads to a bias in perceived spatial frequency. As mentioned earlier, log likelihood functions are asymmetrical around the veridical grating frequency in the presence of low-pass and high-pass filtered noise. When these log likelihood functions are integrated with broad read-out functions, the asymmetry biases perceived spatial frequency towards lower and higher spatial frequencies in low-pass and high-pass filtered noise (Figure 4).

The log likelihood ratio can be obtained through weighted summation using a specific weighting profile (see supporting texts S1.2.2 and S1.3.2). Figure 6b shows the weighting profiles for discrimination between a grating of 1 c/deg and 5.5 c/deg that are consistent with the read-out functions provided in Figure 6a. High-precision read-out corresponds to precision pooling, i.e., preferentially weighting neurons that are tuned to either 1 c/deg or 5.5 c/deg (broken lines). In case of low-precision read-out, considerable weight is assigned to neurons that are insensitive to the two relevant frequencies (full lines).

The read-out function displayed in Figure 6a is equivalent to the signal template provided in Figure 6c. While the template resembles the ideal template of a noise-free grating, it additionally contains a considerable amount of irrelevant spatial frequency components. The template thus closely matches the actual noise-embedded gratings presented during the experiment.

### Comparison of empirical and ideal observer performance

To evaluate the performance of an ideal observer implementing optimal decoding, we reduced the width of the model's read-out functions to an infinitely small value while keeping all other parameters at the best-fitting values. Under these conditions, the predicted perceptual bias disappears and discrimination performance increases from 84% to 95% correct (Figure 1b, right panel). Even for read-out function widths as broad as 1.5 octaves, the model predicts nearly unbiased discrimination performance and reaches 94% correct on average. This indicates that a considerable amount of spatial frequency information is encoded in the V1 population response, even in the presence of filtered visual noise, but that this information is not used to maximize performance.

Ideal observer performance may match empirical performance when a lower amount of information is assumed in the encoding stage. To test this possibility, we constrained read-out function width to an infinitely-small value but refitted all other model parameters. Excitatory receptive field bandwidth increased from 0.9 octaves in the unconstrained model to 1.9 octaves while the bandwidth of the inhibitory gain mechanism increased to 5.5 octaves. The spontaneous discharge rate in broadband noise is now estimated at 26 Hz instead of 5.3 Hz. The model thus reacts to the optimal decoding constraint by reducing the amount of spatial frequency information encoded in the simulated population response. The constrained model manages to predict the bias in the discrimination task to a reasonable degree (Figure 2, broken lines). However, as a result of the broad spatial frequency tuning and strong noise excitation, the model considerably overestimates the degree of masking in the notched noise condition and to a lesser extent in the broadband noise condition (Figure 3, broken lines). Overall goodness-of-fit of the constrained model is significantly lower compared to the unconstrained model (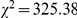, parametric Monte-Carlo test, 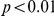) and the Weibull model (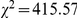, parametric Monte-Carlo test, 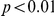). Additionally, we evaluated an ideal observer model in which optimality is assumed for contrast detection while unconstrained read-out functions are implemented for discrimination. Goodness-of-fit of this model approximated the goodness-of-fit of the fully unconstrained model (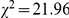, parametric Monte-Carlo test, 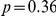), suggesting that the possibility of optimal read-out in the contrast detection task cannot be excluded based on our data.

**Figure 7:**
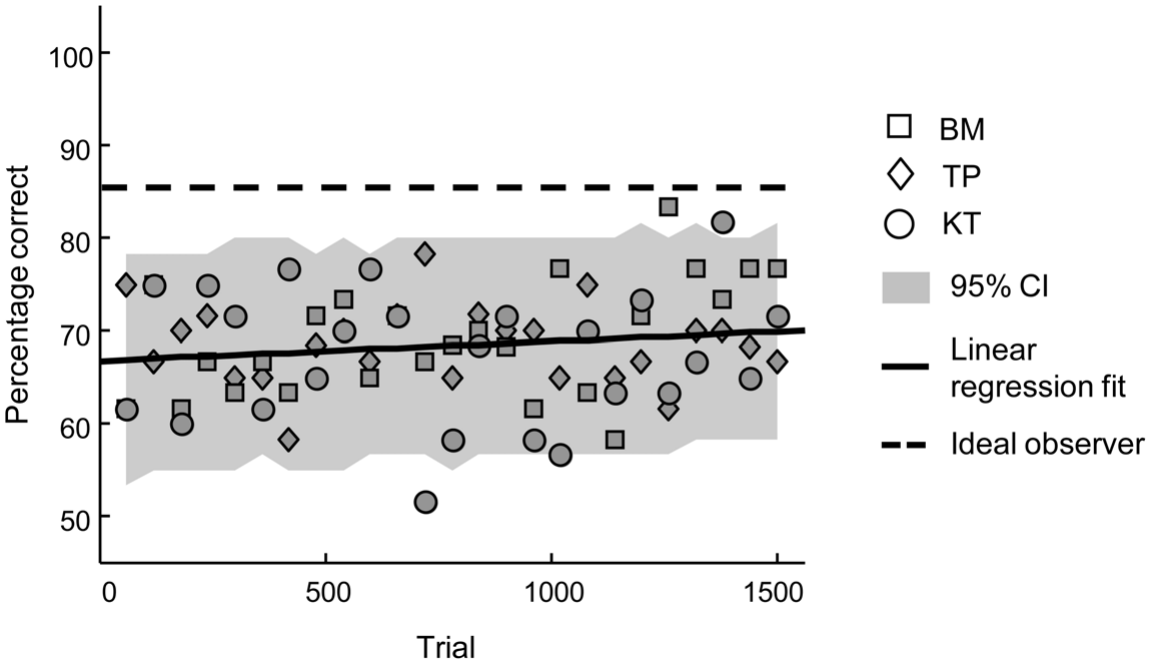
Results of the control experiment. Symbols indicate the pooled data of individual observers. The full line indicates the linear regression fit. The broken line denotes the performance of the ideal observer.

A possible issue concerns the relatively high performance achieved by our observers in the discrimination task. Despite being significantly biased, observers reached an average performance of 84.5% correct (binomial 95% CI = [0.839, 0.851]). Such high performance might discourage the use of an unbiased yet possibly more demanding decoding strategy. Furthermore, the performance gain that can be obtained using a more optimal decoding scheme is limited. As the observed high performance is due to a considerable number of unbiased stimulus conditions, we conducted a similar discrimination experiment and only included stimulus conditions in which the bias was particularly strong (see methods). Observers reached an average performance of 68% correct in this control experiment. This value is significantly lower than the performance of 86% correct predicted by the ideal observer (parametric Monte-Carlo test, 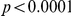). Furthermore, performance did not increase significantly over trials (linear regression slope = 0.002%, parametric Monte-Carlo test, 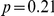), suggesting that suboptimal performance in our experiments was not due to a lack of training (Figure 7).

## Discussion

In the present study, we report a new perceptual bias. Filtered visual noise significantly alters the perception of two known gratings in a two-alternative spatial frequency discrimination task, shifting perceived spatial frequency to lower and higher frequencies in respectively low-pass and high-pass filtered noise conditions. The fact that we observed such a strong bias is not self-evident. Observers received extensive training prior to the start of the experiments and auditory feedback was provided after each trial. Furthermore, we informed observers of the veridical grating spatial frequencies via noise-free grating templates presented at the beginning of each trial block. It should be noted that the bias cannot be attributed to merely a low signal-to-noise ratio, preventing observers from detecting the gratings when embedded in visual noise. Gratings were presented at relatively high contrasts in the discrimination experiment, corresponding to the 84%-correct detection thresholds measured in broadband noise. Moreover, the fact that we measured high overall performance and correspondingly steep psychometric functions in the discrimination task indicates that observers base their decisions -at least partially- on the veridical grating spatial frequency.

We propose a population code model, consisting of a physiologically inspired encoding front-end followed by a simple linear decoder. The best-fitting model accurately predicts the perceptual bias in the discrimination task while simultaneously capturing contrast detection performance in the absence of visual noise, in broadband noise as well as in filtered notched noise using a single set of parameters. Although few constraints were imposed, the estimates of the encoding stage parameters lie close to values reported in macaque or cat V1 single-cell recording studies. Ideal observer analysis revealed that a considerable amount of spatial frequency information is available in the encoding stage, even in the presence of filtered visual noise. Furthermore, using this information to achieve unbiased spatial frequency discrimination does not require complex decoding. A biologically plausible linear decoder [12], only requiring knowledge of V1 spatial frequency tuning but adopting narrow read-out functions to transform the likelihood function into a likelihood ratio decision variable, can achieve unbiased discrimination performance. In principle, however, a model implementing optimal read-out functions can account for the bias in the discrimination task, provided that the amount of information assumed to be available in the encoding stage is limited. The perceptual bias is thus a hallmark of suboptimal spatial frequency processing, which can be due to either inefficiencies in the encoding or decoding stage. However, grating detectability tightly constrains the amount of information available in the encoding stage, which allowed us to attribute the perceptual bias to suboptimal decoding involving broad read-out functions.

While our unified suboptimal likelihood read-out scheme for contrast detection and spatial frequency discrimination accounts for all behavioural measurements, our data are not inconsistent with optimal read-out in the contrast detection task. However, previous studies [34] comparing human to ideal contrast detection performance have reported low detection efficiencies, which may be consistent with suboptimal likelihood read-out. Furthermore, it seems unlikely that observers, while able to read out the likelihood function optimally in the detection task, would not manage to do so in the discrimination task. One possibility is that observers do not use a likelihood ratio to discriminate between spatial frequencies. For instance, observers may estimate grating spatial frequency and indicate which of two gratings yields the largest estimate. Previous studies [35], [36] have proposed maximum-likelihood, maximum-a-posteriori (MAP) or minimum mean squared error (MMSE) decoding to estimate unknown stimulus quantities from the population response. In our task, however, maximum-likelihood decoding will yield unbiased estimates of grating frequency in the presence of filtered noise. While filtered noise induces an asymmetry in the likelihood function, the location of its peak remains largely unchanged (Figure 4). The MAP and MMSE decoders would estimate grating spatial frequency based on respectively the maximum and the mean of the posterior probability function. The posterior is obtained by integrating the likelihood function with a prior probability function. In a two-alternative discrimination task, only two grating spatial frequencies are a-priori likely to occur. Therefore, the prior probability of these specific frequencies equals 0.5 while the prior probability of other, irrelevant frequencies equals zero. Integrating the likelihood function with such an optimal prior to obtain the posterior is equivalent to reading out the likelihood function using infinitely-small and appropriately-placed read-out functions. Consequently, decoders operating on the posterior are equivalent to our decoder computing a likelihood ratio decision variable with high precision and fail to predict the perceptual bias (see supporting text S1.4). An interesting hypothesis is that observers do not use a prior that is optimal to the task at hand, but instead rely on a general prior based on natural scene statistics [36]. As the spatial frequency spectrum of natural scenes can be characterised by a 1/f relationship between amplitude and spatial frequency [37], this prior would presumably be broadband and skewed towards low spatial frequencies. A MAP or MMSE estimator incorporating such a prior might also predict a noise-induced perceptual bias. This bias, however, will be asymmetric as likelihoods of low spatial frequencies are preferentially weighted when obtaining the posterior. More specifically, low-pass filtered noise is expected to induce a stronger bias compared to high-pass noise. Our data provide no consistent evidence for such an asymmetry (see supporting text S2).

To read out the likelihood function with high precision, the visual system requires a-priori knowledge of the grating spatial frequencies presented on each trial in the discrimination experiment. In other words, the brain has to be able to represent appropriate, narrow signal templates. A theoretical issue concerns whether and how such templates can be reliably implemented in populations of noisy and broadly-tuned neurons. Recurrent network models have been proposed, capable of removing noise from the population response and representing arbitrarily small templates with high accuracy [38], [39]. Our results indicate that observers did not have access to such accurate templates and may have been uncertain about grating spatial frequency. Signal uncertainty has been studied extensively in psychophysical detection tasks [19], [40] but not in the context of neural population decoding [41].

Our model differs from traditional psychophysical models of early visual encoding of gratings embedded in external noise [42], as these models typically assume that external noise only affects the variability of sensory responses without changing the average response (see supporting text S4). Physiological evidence, however, suggests that response variability is proportional to response mean, even in the presence of early additive noise [43]. Furthermore, in our discrimination experiment, changes in sensory variability alone may affect the slope of the psychometric functions but cannot account for systematic shifts in their position [36]. Moreover, similar physiologically inspired population code models have been successfully used to relate single-cell response characteristics to human psychophysical grating detectability and discriminability [29], [44].

Various studies have measured psychophysical two-alternative detection and discrimination performance while simultaneously determining the amount of sensory information encoded by early visual brain areas. A common finding, consistent with our results, is that simple decoding schemes, while straightforward to implement and achieving near-optimal performance, consistently overestimate empirical performance [8]–[11]. An important question that remains to be answered is how these results can be reconciled with studies reporting near-optimal population decoding and precision pooling in two-alternative discrimination tasks. The answer may lie in the complexity and predictability of stimuli used in different tasks. For instance, Purushotaman and Bradley [7] report precision pooling in a fine orientation discrimination task using highly-coherent and thus highly-predictable random dot stimuli. Shadlen et al. [11] and Cohen and Newsome [9], reporting a lack of precision pooling, used coarse discrimination tasks in which the average coherence of the random dot stimuli was considerably lower. We also used highly-stochastic broadband stimuli in our experiments, which might have prevented the formation of small-band signal templates.

Perceptual biases are usually interpreted as a hallmark of optimal statistical inference [36]. Here, we developed a novel paradigm to obtain a set of findings that challenge this view. Although we focused on grating detection and discrimination, the utility of our approach is not limited to the domain of pattern vision. Future studies may investigate whether a similar bias can be found in other tasks such as fine motion orientation discrimination, often used to demonstrate optimal population decoding. A crucial question for future research will be to identify determinants that explain the large variations in sensory decoding efficiency across different stimulus domains and tasks.

## Methods

### Psychophysics

#### Equipment

Stimuli were presented on a carefully linearized monochrome Siemens SMM 21106 LS monitor with white phosphor (P-45). Spatial resolution was 996×777 pixels at 130 Hz and 8-bit luminance precision was obtained at each contrast level used in the experiments. Viewing distance was 124 cm, corresponding to a pixel size of 0.0181 degrees of visual angle. The experiment was run in a darkened room, with the display's mean background luminance equal to 47.5 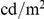.

#### Observers

Three observers (TP, BM and KT) participated in two spatial frequency discrimination experiments. Two observers (RV and NV) participated in a contrast detection experiment. All observers but TP were naive to the purpose of the experiments. Observers received extensive training prior to data collection and had normal or corrected-to-normal vision.

#### Stimuli

Stimuli were Gabor gratings, consisting of a horizontally-orientated sinusoidal grating subtending 9.2 degrees of visual angle, multiplied by a 2-D circularly-symmetrical Gaussian envelope with a standard deviation of 3.7 degrees of visual angle. A set of 1-D horizontally-orientated noise images was generated by sampling pixel luminance values from a Gaussian distribution centred at mean luminance. Mean noise-power density equalled 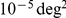. The noise-power density spectrum of broadband noise images was flat to an upper bound of 27.4 c/deg. Low-pass and high-pass filtered noise images were obtained by removing spatial frequency components respectively above or below 5.5 c/deg. We obtained notched noise images by removing spatial frequency components within a 4-octave-wide notch around 5.5 c/deg. Grating and noise images were presented on alternating frames without visible flicker. A fresh noise sample was used for each stimulus presentation.

#### Spatial frequency discrimination experiments

A two-alternative two-interval forced-choice (2-AFC) procedure was used to measure spatial frequency discrimination performance in the presence of broadband and filtered visual noise. On each trial, a fixation cross was presented for 250 ms which disappeared 500 ms before the onset of auditory-cued 50 ms stimulus intervals. One interval contained a grating of 5.5 c/deg (the standard grating). The other interval contained a grating of variable spatial frequency (the comparison grating). Observers indicated the interval containing the grating of the highest spatial frequency. Auditory feedback was provided after each trial. In the main conditions, the standard grating was embedded in low-pass or high-pass filtered noise with a cut-off at 5.5 c/deg while the comparison grating was presented in broadband noise. Control conditions in which comparison instead of standard grating was embedded in filtered noise were included to prevent the use of the noise conditions as discrimination cues. In addition, we included two conditions in which both gratings were presented either in broadband noise or in the absence of noise. All noise conditions were randomly intermixed. High-contrast, noise-free templates of standard and comparison gratings were presented at the beginning of each block of 50 trials, within which comparison spatial frequency was kept constant. Eight comparison frequencies, sampled logarithmically between 1.9 and 15.6 c/deg, were tested in a first discrimination experiment. The relative matching frequency, corresponding to the comparison frequency yielding a choice proportion of 50%, was determined by fitting cumulative Weibull psychometric functions using a maximum-likelihood fitting procedure [45]. Confidence intervals were determined using a parametric Monte-Carlo bootstrap procedure [46]. To ensure a constant and sufficiently high signal-to-noise ratio across grating spatial frequencies, each grating was presented at the contrast level corresponding to the 84% detection threshold in broadband noise, estimated separately for each observer in a preliminary contrast detection experiment (see supporting text S3). Each observer completed a total of 4800 trials. We conducted a second discrimination experiment, only including one comparison condition in which a strong bias was observed (observer BM and TP: 4.7 c/deg, KT: 6.4 c/deg). Observers completed 250 trials per experimental condition, yielding a total of 1500 trials. Proportion correct was calculated for each subset of 60 trials. A linear function relating proportion correct to subset index was fitted to the data using least-squares regression.

#### Contrast detection experiment

A similar 2-AFC procedure was used to measure contrast detection performance in the absence of visual noise and in the presence of broadband and notched noise. In the no-noise condition, one interval contained a blank while the target interval contained a grating of 5.5 c/deg, i.e., the spatial frequency of the standard grating in the discrimination task as well as the average spatial frequency of all gratings presented in the discrimination experiments. In the noise conditions, both intervals contained a different background noise image. Observers were instructed to indicate the target interval and received auditory feedback after each trial. The grating was presented at one of sixteen (in the no-noise and broadband noise conditions) or nine (in the notched noise condition) fixed contrast levels. Noise and contrast conditions were randomized across trials. A high-contrast, noise-free template was presented at the beginning of each trial block. Cumulative Weibull psychometric functions were fitted to the data using a maximum-likelihood fitting procedure [45]. Confidence intervals were determined using a parametric Monte-Carlo bootstrap procedure [46]. Each observer completed a total of 4100 trials.

### Model

#### Encoding stage

V1 population responses to gratings embedded in filtered visual noise were derived following the logic of the normalization model of simple cells [20]. The average response of an individual neuron *i* to a sinusoidal grating of spatial frequency 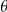 and contrast *c* embedded in noise background *N* is given by the following equation:
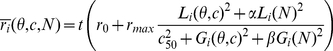
(1)where 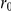 is the spontaneous discharge rate of the neuron (in Hertz), 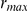 the maximal firing rate (in Hertz) and 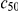 the semi-saturation constant. *t* equalled the stimulus presentation time (in seconds). 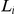 equals the response of the linear excitatory receptive field of neuron *i*, while 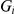 denotes the linear response of the broadly-tuned contrast gain control pool inhibiting the responses of neuron *i*. These responses are obtained using a linear convolution of the 1-D spatial frequency amplitude spectrum of a grating or (filtered) noise background and a Gaussian-shaped tuning function. It should be noted that we normalized the linear filter responses to a full-contrast grating to equal one across all grating spatial frequencies (see supporting text S3). The main effects of visual noise on the contrast response function, i.e., an excitatory increase in spontaneous discharge and an inhibitory shift towards higher contrasts and lower response rates, are respectively captured by 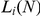 and 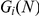. The absolute size of these effects are controlled by two scaling parameters 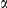 and 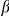. Notably, we assumed separate pathways for the processing of signal and noise to obtain a highly-flexible descriptive model rather than functional model of visual noise effects. As physiological data describing these effects are scarce, we do not impose strong a-priori constraints on the relative size of excitatory and inhibitory responses to signal and noise.

Response variance, known to scale with mean response rate [47], is defined as:
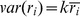
(2)where k is the proportionality constant. As implementing the full covariance matrix is computationally prohibitive, we use a diagonal covariance matrix in our simulations, thus effectively ignoring interneural correlations. Such correlations have been observed in many studies [9], [48]. For primary visual cortex, most estimates lie between 10% and 15%. Incorporating (limited-range) correlations of that magnitude in our model merely rescales the overall signal-to-noise ratio of the population code and limits the amount of information gained by increasing population size beyond approximately 100 neurons. These effects were closely approximated in our model by limiting population sizes to 100 uncorrelated neurons and including an overall efficiency parameter in the decoding stage.

#### Decoding stage

We implemented a biologically plausible log likelihood ratio decoder that assumes independent Poisson noise and is ignorant about the effect of the filtered noise on the population response. The log likelihood function of spatial frequency 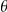 obtained by this decoder is provided by:
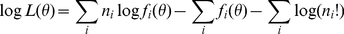
(3)where 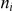 equals the response and 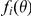 denotes the spatial frequency tuning function of neuron *i*. In supporting text S1.1, we provide the full derivation of this equation and show that only the first term of the right hand side is relevant to the decision variable in two-alternative two-interval grating detection and discrimination tasks. We therefore define the reduced log likelihood function as:
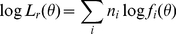
(4)The log likelihood of a specific spatial frequency 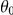, denoted 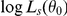, is obtained by integrating the reduced likelihood function with a Gaussian-shaped read-out function 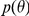 centred at 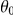:
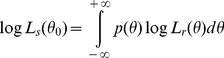
(5)In the optimal log likelihood ratio decoder, 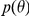 is infinitely small (see supporting text S1.2). This decoder is able to compute the log likelihood of 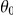 with infinitely-high precision. We include read-out function bandwidth as a free parameter. Our decoder approximates the optimal decoder when the bandwidth parameter approaches zero. For large bandwidths, the decoder is suboptimal and computes the log likelihood of 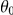 with low precision, averaging the likelihood of a broad range of spatial frequencies (see supporting text S1.3).

In our two-alternative two-interval spatial frequency discrimination task, gratings of high and low spatial frequency are presented on each trial. A log likelihood ratio is used to discriminate between these two frequencies. The log likelihood ratio for interval 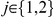 is defined as:
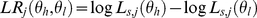
(6)where 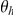 and 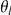 equal the high and low grating spatial frequency presented during the trial. A log likelihood ratio is computed for each interval. When instructed to indicate the interval containing the high spatial frequency grating, the decoder will select the interval yielding the highest log likelihood ratio. The resulting decision variable is given by:
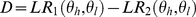
(7)A similar decision variable was defined for the grating detection task (see supporting text S1.2.1). The derivation of predicted choice proportion and proportion correct are provided in supporting text S1.2.2.

From equation 4, one can see that the (reduced) log likelihood can be obtained through linear precision pooling. To compute the log likelihood of spatial frequency 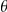, the response of each neuron is weighted by the sensitivity of that neuron to 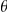. This sensitivity is captured by the logarithm of the neuron's spatial frequency tuning function evaluated at 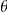. The log likelihood of a spatial frequency thus only depends on the responses of neurons tuned to that frequency. Likewise, the ratio of the log likelihoods of two grating spatial frequencies only depends on the responses of two subpopulations of neurons, each tuned to one of the grating frequencies. Notably, such precision pooling requires the likelihood function to be read out with infinitely-high precision. When the likelihood function is read out with lower precision following equation 5, the likelihood of a spatial frequency 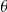 will depend on the responses of a broader subpopulation of neurons, including neurons that are insensitive to 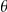. The neural implementation of the log likelihood ratio decoder through linear weighted summation and precision pooling is described in detail in supporting texts S1.2.2 and S1.3.2.

#### Model constraints and fitting

A limited number of parameters were fixed at physiologically plausible values. Our data did not provide sufficient constraints to estimate these parameters. Most importantly, changing the exact values of these parameters does not affect the conclusions of the present study. More specifically, 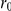 was set to 1% of the maximal response [49] and 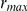 equalled 100 Hz [21]. Neuron preferred spatial frequencies were sampled uniformly between 0.25 and 60 c/deg. The proportionality constant *k* determining response variance was set to 1.5 [50]. Thus, external noise only indirectly affects response variance through changes in the mean response, which is in agreement with recent physiological evidence [43] (see supporting text S4). Noise-power density equalled the actual value used in the experiments. Discrimination performance was simulated at the average grating contrast used in the discrimination experiments for computation convenience (see supporting text S3). All other model parameters were included as free parameters. The efficiency parameter was constrained between 0 and 1. A maximum-likelihood fitting procedure [45] was used to fit the model simultaneously to the contrast detection and spatial frequency discrimination datasets. The deviance statistic, expressing the ratio between the likelihood of the model under consideration and a saturated model with no residual error, was calculated for each dataset. The parameter combination minimizing total deviance was determined using an implementation of the Nelder-Mead simplex algorithm. Multiple fits were performed using random initial parameter values. Confidence intervals on parameter estimates were determined using parametric Monte-Carlo bootstrap procedures [46].

## Supporting Information

Figure S1Weighting profile 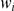 in case of two-alternative detection of a grating spatial frequency 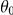 (left) and two-alternative discrimination of grating spatial frequencies 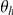 and 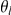 (right). Vertical lines denote the grating spatial frequencies. It can be seen that a log likelihood ratio decoder, selectively evaluating the likelihood of relevant spatial frequencies, preferentially weights neurons tuned to these frequencies and ignores neurons tuned to other frequencies. The best-fitting parameter values reported in the main text were used to specify the encoding front-end.(TIF)

Figure S2Off-looking when discriminating two close-together grating spatial frequencies 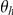 and 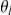. Neurons tuned slightly away from 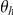 and 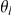 are preferentially weighted. The weighting profile 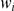 approximates a difference of two Gaussian functions centred at 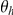 and 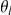. As a result of the subtraction and because these functions are not infinitely narrow, the peak and trough of the weighting profile are shifted away from 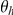 and 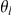. The best-fitting parameter values reported in the main text were used to specify the encoding front-end.(TIF)

Figure S3Relationship between likelihood read-out precision and precision pooling. Left: read-out functions used to obtain the discrete likelihood of spatial frequency 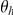. Right: corresponding weighting profiles when discriminating between grating spatial frequencies 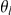 and 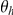. The best-fitting parameter values reported in the main text were used to specify the encoding front-end.(TIF)

Figure S4 Individual data for the discrimination task. Data of the main and control conditions are provided respectively in the top and bottom row. Red, green and blue colors respectively denote low-pass filtered, white and high-pass filtered noise conditions. Full lines represent the best-fitting Weibull psychometric functions.
(TIF)

Figure S5 Individual data for the detection task. Dark gray, light gray and green colors respectively denote the no-noise, notched noise and white noise conditions. Full lines represent the best-fitting Weibull psychometric functions.
(TIF)

Figure S6 84%-correct detection thresholds in broadband noise for different grating spatial frequencies. Full lines indicate the best-fitting second-degree polynomial contrast sensitivity functions. Blue, green and red colors respectively represent the data of subject KT, BM and TP. The broken line denotes the average contrast used in the discrimination tasks. This contrast was used to simulate discrimination performance.
(TIF)

Figure S7 Predicted discrimination performance for the constrained model (left panel) and unconstrained model (right panel) when a Fano factor of 1 (dotted lines) instead of 1.5 (full lines) was assumed. Other parameters were kept at the best-fitting values.
(TIF)

Figure S8 Predicted detection performance for the constrained model (left panel) and unconstrained model (right panel) when a Fano factor of 1 (dotted lines) instead of 1.5 (full lines) was assumed. Other parameters were kept at the best-fitting values.
(TIF)

Text S1 Supporting text providing a detailed description of the population decoding model.
(PDF)

Text S2 Supporting text discussing inter-individual differences and data pooling.
(PDF)

Text S3 Supporting text discussing contrast sensitivity across spatial frequency.
(PDF)

Text S4 Supporting text discussing the role of across-trial neural response variability.
(PDF)
