# Supplementary figures and images for: A New Perceptual Bias Reveals Suboptimal Population Decoding of Sensory Responses
Source: PLoS Comput Biol. 2012 Apr 12;8(4):e1002453. doi: 10.1371/journal.pcbi.1002453 (PMC3325184; doi:10.1371/journal.pcbi.1002453)

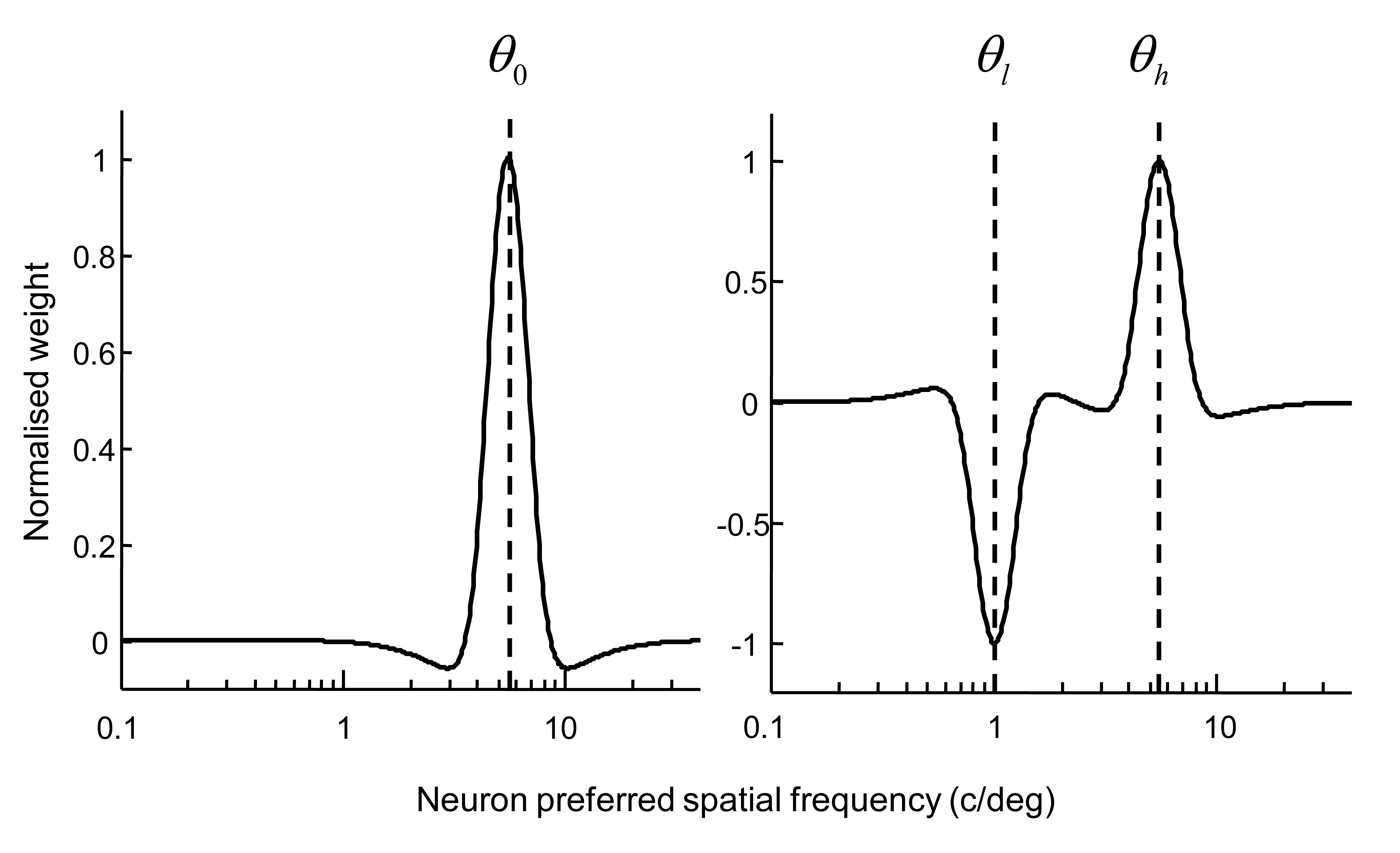

Supplement: Figure S1 — Weighting profile in case of two-alternative detection of a grating spatial frequency (left) and two-alternative discrimination of grating spatial frequencies and (right). Vertical lines denote the grating spatial frequencies. It can be seen that a log likelihood ratio decoder, selectively evaluating the likelihood of relevant spatial frequencies, preferentially weights neurons tuned to these frequencies and ignores neurons tuned to other frequencies. The best-fitting parameter values reported in the main text were used to specify the encoding front-end. (TIF) [file pcbi.1002453.s001.tif]

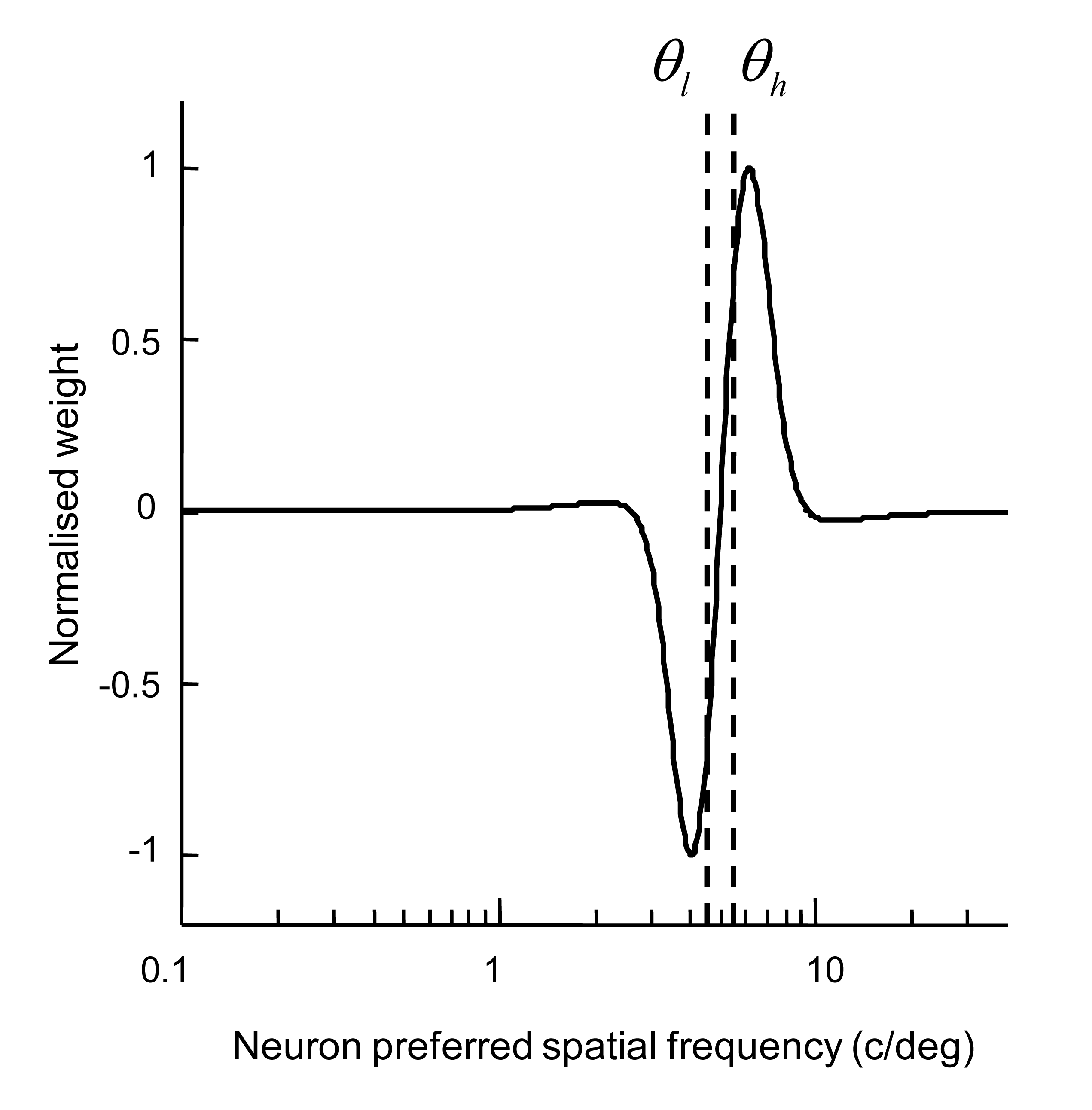

Supplement: Figure S2 — Off-looking when discriminating two close-together grating spatial frequencies and . Neurons tuned slightly away from and are preferentially weighted. The weighting profile approximates a difference of two Gaussian functions centred at and . As a result of the subtraction and because these functions are not infinitely narrow, the peak and trough of the weighting profile are shifted away from and . The best-fitting parameter values reported in the main text were used to specify the encoding front-end. (TIF) [file pcbi.1002453.s002.tif]

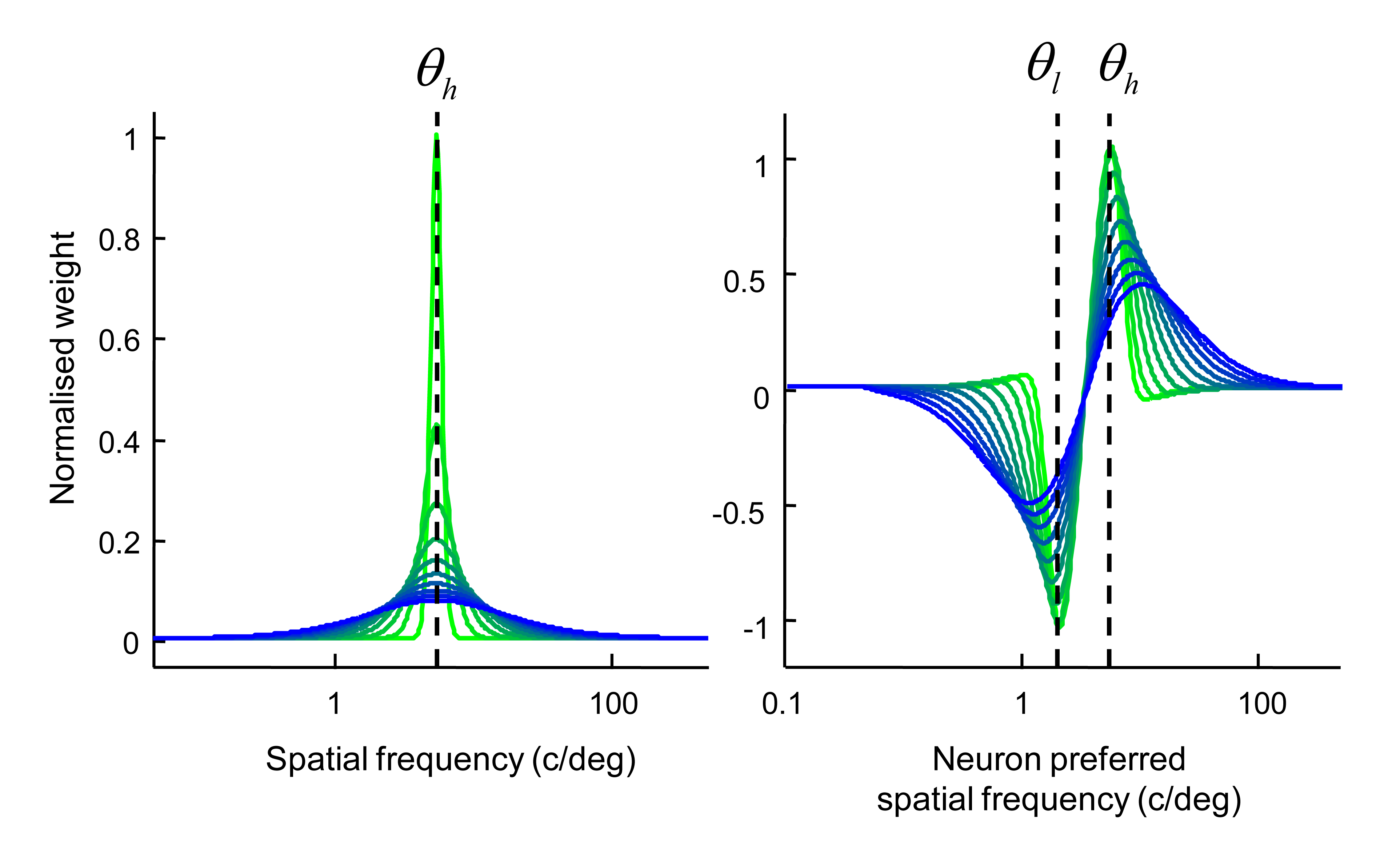

Supplement: Figure S3 — Relationship between likelihood read-out precision and precision pooling. Left: read-out functions used to obtain the discrete likelihood of spatial frequency . Right: corresponding weighting profiles when discriminating between grating spatial frequencies and . The best-fitting parameter values reported in the main text were used to specify the encoding front-end. (TIF) [file pcbi.1002453.s003.tif]

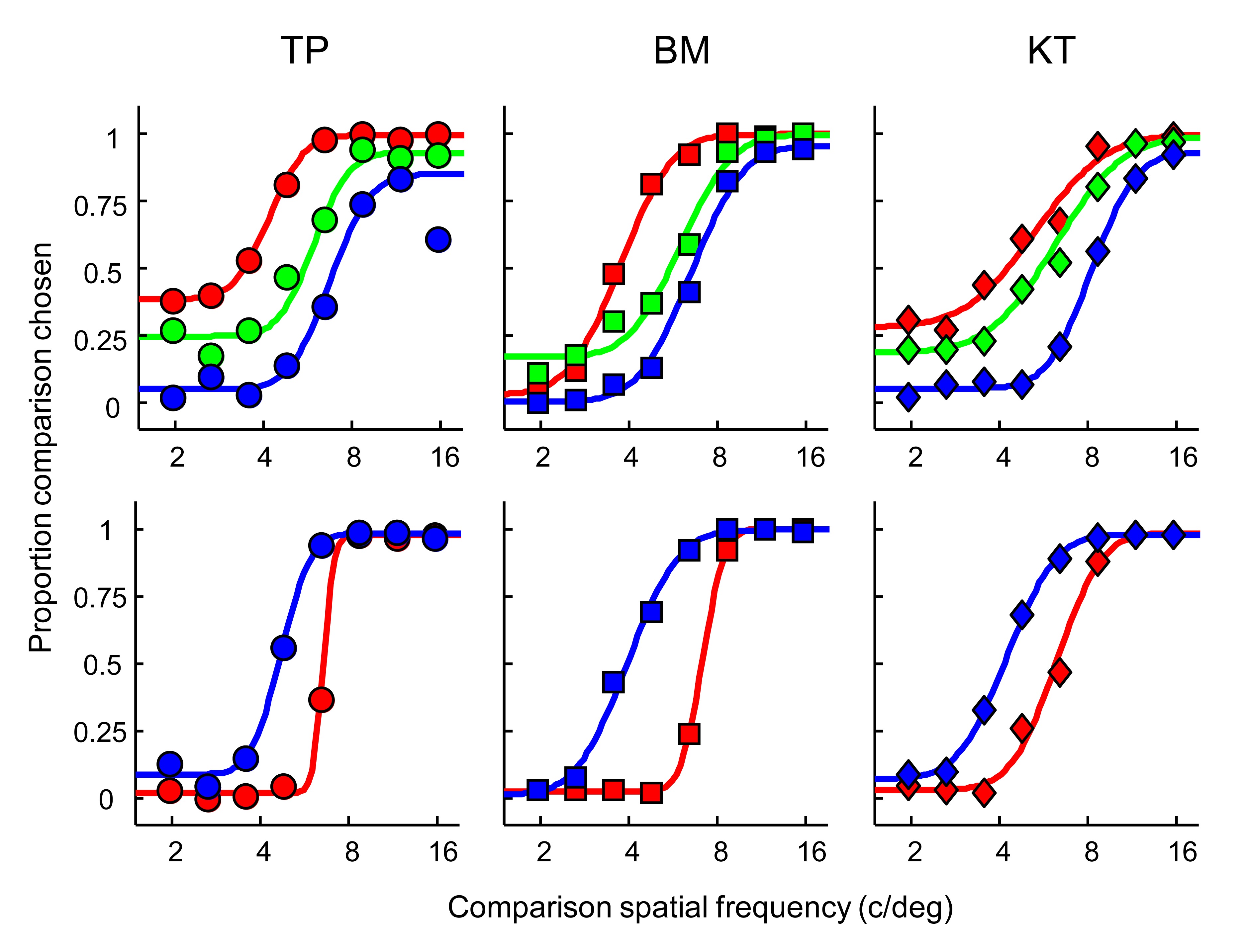

Supplement: Figure S4 — Individual data for the discrimination task. Data of the main and control conditions are provided respectively in the top and bottom row. Red, green and blue colors respectively denote low-pass filtered, white and high-pass filtered noise conditions. Full lines represent the best-fitting Weibull psychometric functions. (TIF) [file pcbi.1002453.s004.tif]

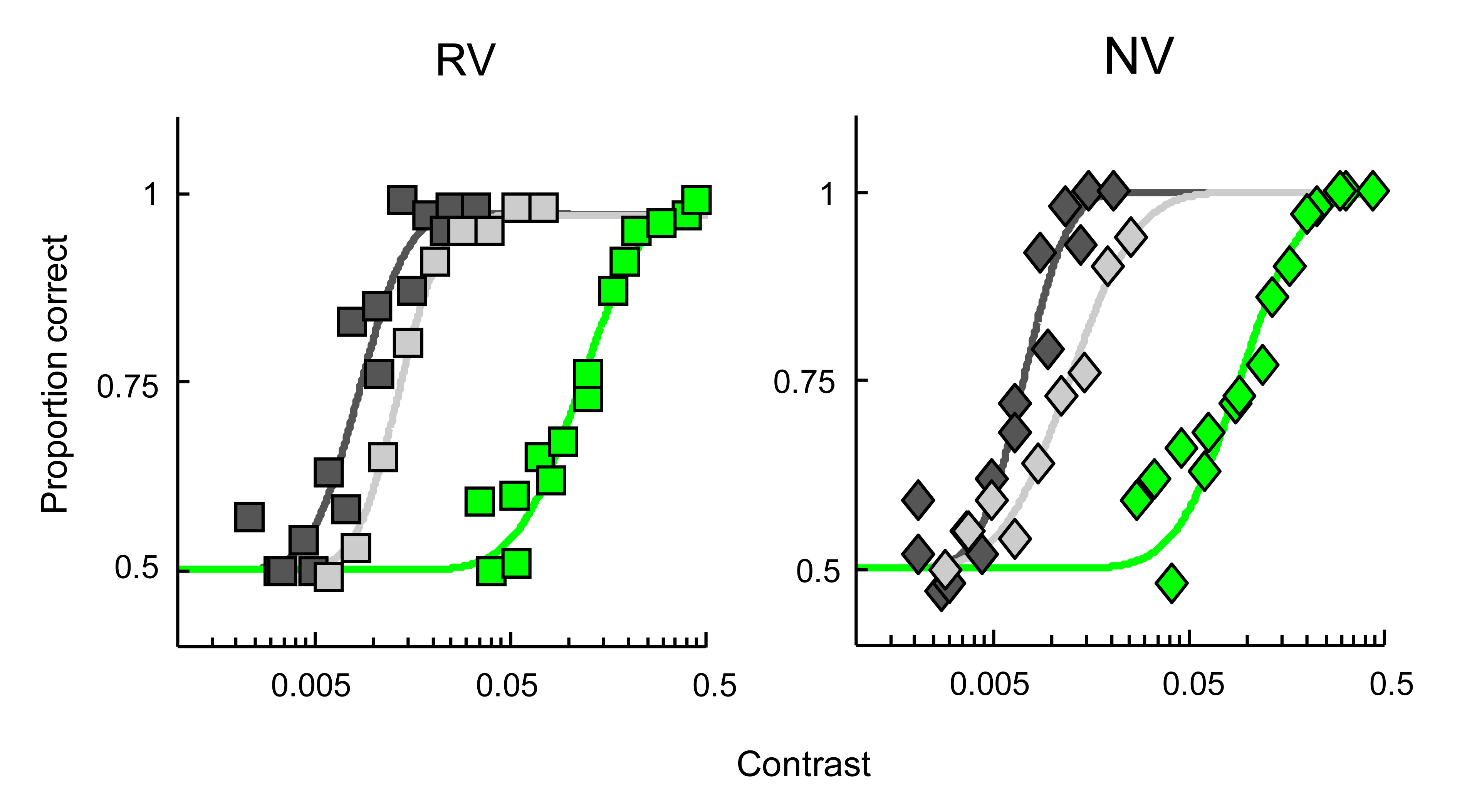

Supplement: Figure S5 — Individual data for the detection task. Dark gray, light gray and green colors respectively denote the no-noise, notched noise and white noise conditions. Full lines represent the best-fitting Weibull psychometric functions. (TIF) [file pcbi.1002453.s005.tif]

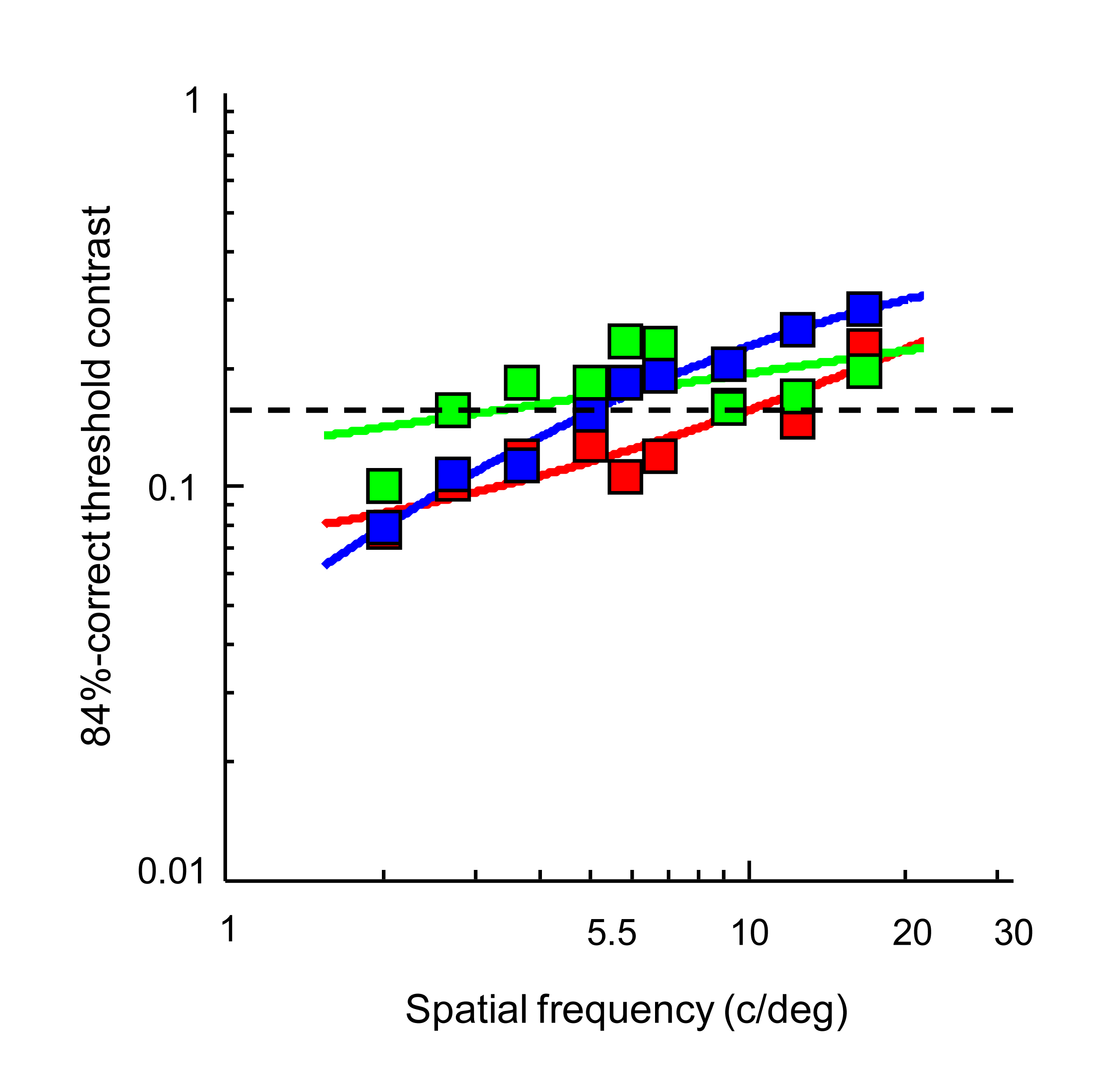

Supplement: Figure S6 — 84%-correct detection thresholds in broadband noise for different grating spatial frequencies. Full lines indicate the best-fitting second-degree polynomial contrast sensitivity functions. Blue, green and red colors respectively represent the data of subject KT, BM and TP. The broken line denotes the average contrast used in the discrimination tasks. This contrast was used to simulate discrimination performance. (TIF) [file pcbi.1002453.s006.tif]

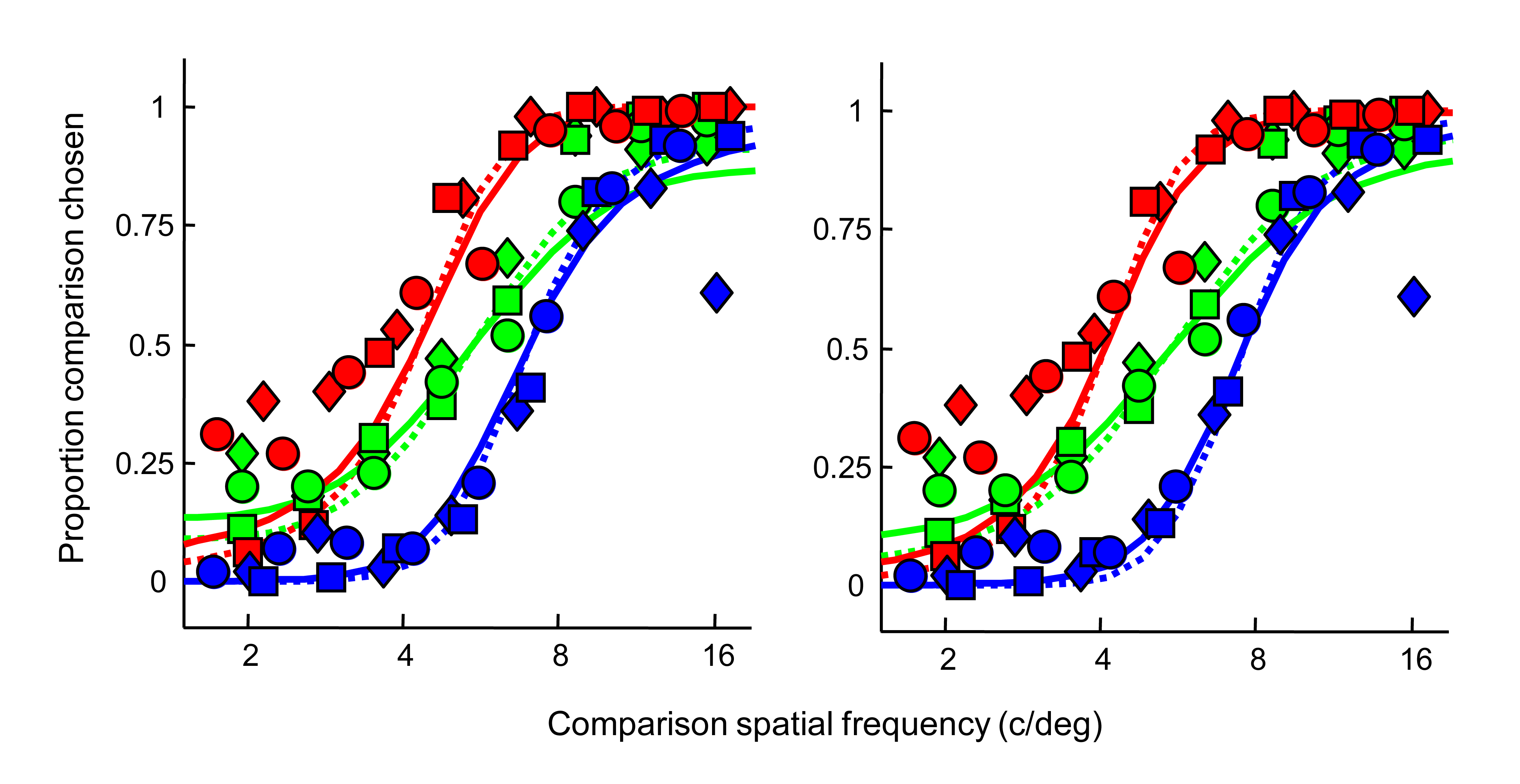

Supplement: Figure S7 — Predicted discrimination performance for the constrained model (left panel) and unconstrained model (right panel) when a Fano factor of 1 (dotted lines) instead of 1.5 (full lines) was assumed. Other parameters were kept at the best-fitting values. (TIF) [file pcbi.1002453.s007.tif]

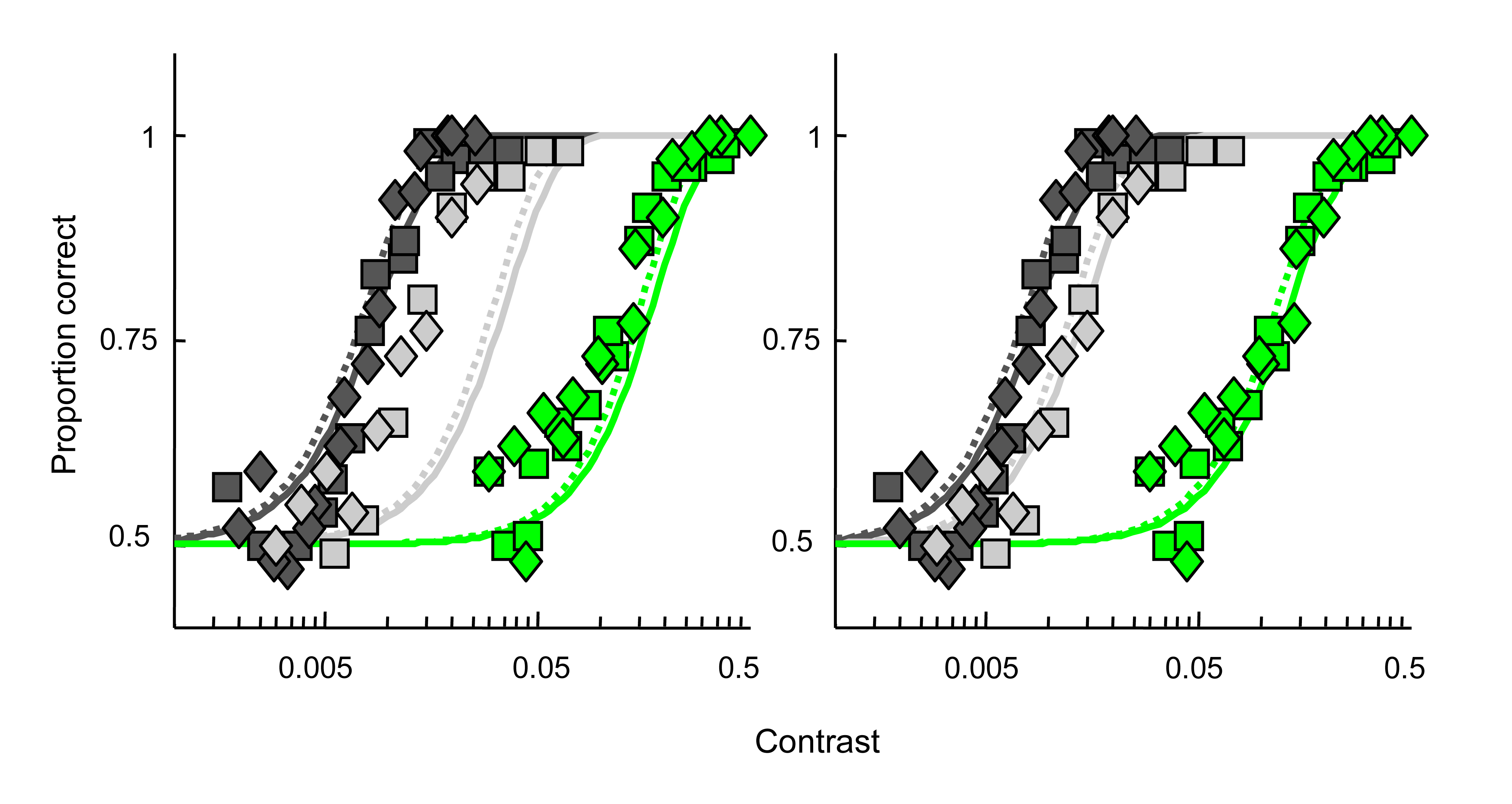

Supplement: Figure S8 — Predicted detection performance for the constrained model (left panel) and unconstrained model (right panel) when a Fano factor of 1 (dotted lines) instead of 1.5 (full lines) was assumed. Other parameters were kept at the best-fitting values. (TIF) [file pcbi.1002453.s008.tif]
