# Supplementary material for: A New Perceptual Bias Reveals Suboptimal Population Decoding of Sensory Responses
Source: PLoS Comput Biol. 2012 Apr 12;8(4):e1002453. doi: 10.1371/journal.pcbi.1002453 (PMC3325184; doi:10.1371/journal.pcbi.1002453)
Supplement: Text S1 — Supporting text providing a detailed description of the population decoding model. (PDF) [file pcbi.1002453.s009.pdf]

Supporting text S1: modeling population decoding

1.1 Deriving the likelihood function of grating spatial frequency

The likelihood of a grating of spatial frequency θ, contrast c embedded in a noise background N is defined as the probability of observing a response n_i of neuron i given the presentation of that grating. Under the assumption of Poisson variability, likelihood is defined by:

$$l_i(\theta, c, N) = p\left(n_i|\theta, c, N\right) \quad (1)$$

$$= \frac{\overline{r_i}(\theta, c, N)^{n_i}}{n_i!} e^{-\overline{r_i}(\theta,c,N)} \quad (2)$$

where $\overline{r_i}(\theta, c, N)$ denotes the average response of neuron i to grating of spatial frequency θ and contrast c embedded in noise background N, as specified by equation 4 in the main text. To implement this likelihood computation, the visual system requires knowledge of the high-dimensional function $\overline{r_i}$. In the present study, we consider a simplified one-dimensional likelihood computation that only requires knowledge of a neuron's spatial frequency tuning in the presence of broadband visual input:

$$l_i(\theta) = \frac{f_i(\theta)^{n_i}}{n_i!} e^{-f_i(\theta)} \quad (3)$$

where $f_i(\theta)$ denotes the spatial frequency tuning function of neuron i in the presence of broadband visual noise:

$$f_i(\theta) = \overline{r_i}(\theta, c = 1, N = N_{broadband}) \quad (4)$$

The function defined by equation 3 will hereafter be referred to as the likelihood function of grating spatial frequency. The likelihood that only broadband noise and no grating was presented can be obtained by substituting g for $f_i(\theta)$:

$$g = \overline{r_i}(\theta, c = 0, N = N_{broadband}) \quad (5)$$

It should be noted that g is independent of θ and equal for all neurons specified by our encoding front-end.

Likelihood is often computed in the log space. The log likelihood of θ for the response n_i of neuron i is given by:

$$\log l_i(\theta) = \log \frac{f_i(\theta)^{n_i}}{n_i!} e^{-f_i(\theta)} \quad (6)$$

$$= n_i \log f_i(\theta) - f_i(\theta) - \log(n_i!) \quad (7)$$

Assuming neural responses are statically independent, the log likelihood of θ for the population response can be obtained by summing the log likelihoods of individual responses:

$$\log L(\theta) = \sum_i \log l_i(\theta) \quad (8)$$

$$= \sum_i n_i \log f_i(\theta) - \sum_i f_i(\theta) - \sum_i \log(n_i!) \quad (9)$$

1.2 Optimal log likelihood ratio decoding

1.2.1 Deriving the decision variable

In the two-alternative two-interval spatial frequency discrimination task, two known grating spatial frequencies are presented on each trial. The optimal decoder, maximizing accuracy by minimizing a zero-one

loss function, computes a log likelihood ratio in this task. The log likelihood function is read out at two discrete locations to obtain the log likelihood of the two relevant grating spatial frequencies. The log likelihood ratio for interval j is defined as:

$$LR_j\left(\theta_h, \theta_l\right) = \log L_j\left(\theta_h\right) - \log L_j\left(\theta_l\right) \tag{10}$$

where θ_h and θ_l denote the high and low grating spatial frequency presented during the trial. A log likelihood ratio is computed for each interval. When instructed to indicate the interval containing the high spatial frequency grating, the decoder will select the interval yielding the highest log likelihood ratio. The resulting decision variable is given by:

$$D = LR_1\left(\theta_h, \theta_l\right) - LR_2\left(\theta_h, \theta_l\right) \tag{11}$$

A similar decoder can be specified for the two-alternative two-interval contrast detection task. In this task, a grating of known spatial frequency θ_0 is presented in one of two intervals. The optimal decoder obtains a log likelihood ratio to compare the likelihood that the grating was present to the likelihood that the grating was absent. The former likelihood value is obtained by reading out the log likelihood function at θ_0. The latter likelihood value, denoted as G, can be obtained making use of equation 5 and 9 as explained in the previous section. The log likelihood ratio for interval j is then defined as:

$$LR_j\left(\theta_0\right) = \log L_j\left(\theta_0\right) - \log G_j \tag{12}$$

The log likelihood ratio is computed for each interval. The decoder, instructed to indicate the interval containing the grating, will select the interval yielding the highest log likelihood ratio. The decision variable is provided by:

$$D = LR_1\left(\theta_0\right) - LR_2\left(\theta_0\right) \tag{13}$$

1.2.2 Implementation as linear weighted summation and precision pooling

Due to the subtractions in equations 10-11 and 12-13, the second and third terms of equation 9 cancel out when computing the decision variable D. Therefore, the part of the log likelihood relevant to computing the decision variable is surprisingly simple:

$$\log L(\theta) = \sum_i n_i \log f_i(\theta) \tag{14}$$

This function is referred to as the reduced log likelihood function in the main text. Consequently, the decision variables defined by equation 11 and 13 can be obtained through weighted summation of individual responses:

$$D = \sum_i w_i n_{i1} - w_i n_{i2} \tag{15}$$

where n_{i1} and n_{i2} represent the response of neuron i during the first and second interval. In case of spatial frequency discrimination, the weighting profile w_i is defined as:

$$w_i = \log f_i(\theta_h) - \log f_i(\theta_l) \tag{16}$$

where θ_h and θ_l equal the high and low grating spatial frequency presented during the trial. In case of contrast detection,

$$w_i = \log f_i(\theta_0) - \log g \tag{17}$$

where θ_0 equals the target grating spatial frequency.

Evaluating the (reduced) log likelihood of a specific spatial frequency thus corresponds to summing the responses only of neurons for which the average response differs between the two different conditions. The optimal log likelihood ratio decoder, detecting or discriminating grating frequencies by selectively evaluating the likelihood of these frequencies, adopts precision pooling by preferentially weighting neurons tuned to these frequencies. This can be understood considering that, under the assumption of Poisson variability, neurons with the largest mean response to a stimulus provide the most reliable evidence for the presence of that stimulus. Neurons tuned to other spatial frequencies only contribute noise and should be ignored. Interestingly, if tuning functions are assumed to be Gaussian and of equal bandwidth, the weighting profile w_i defined by equation 17 can be closely approximated by a Gaussian function of neuron preferred spatial frequency, centred at θ_0 with a bandwidth slightly smaller than the tuning function bandwidth (Figure S1, left panel). The weighting profile w_i defined by equation 16 approximates a difference of two such Gaussian functions centred at θ_h and θ_l (Figure S1, right panel).

It should be noted that, following equation 10, log likelihood ratio decoding can be formally described as reading out the continuous log likelihood function at two discrete locations to obtain a likelihood ratio. This does not imply that the log likelihood function is computed explicitly in a first stage prior to read-out in a second stage. The reduced log likelihood of two specific spatial frequencies can be compared directly using the weighting profile defined by equation 16.

Predicted choice proportion and proportion correct are obtained by assuming that D follows a Gaussian distribution with mean and variance provided by:

$$\overline{D} = \sum_i w_i \overline{n_{i1}} - w_i \overline{n_{i2}} \tag{18}$$

and

$$var(D) = \frac{k}{\epsilon} \sum_i w_i^2 \left(\overline{n_{i1}} + \overline{n_{i2}} \right) \tag{19}$$

where k is the proportionality constant defined in equation 2 of the main text and ϵ is a free efficiency parameter. It should be noted that we assume neural noise to be uncorrelated. The mean of the individual responses $\overline{n_i}$ is defined by equation 1 in the main text.

1.2.3 Log likelihood ratio decoding and off-looking

In fine discrimination tasks, i.e., tasks involving discrimination of close-together stimulus values, observers are known to preferentially weight neurons that are slightly tuned away from the relevant stimuli. This strategy has been referred to as off-looking and can be advantageous as so-called off-boundary neurons provide a higher signal-to-noise ratio in fine discrimination tasks. The preferential weighting of off-boundary neurons, however, should not be confused with evaluating the likelihood of off-boundary stimulus values. Even in fine discrimination tasks, only the stimulus values to be discriminated are relevant and only the likelihood of these stimuli should be evaluated. Indeed, the optimal log likelihood ratio decoder, discriminating between two close-together grating spatial frequencies θ_h and θ_l, evaluates the likelihood of *exactly* these frequencies while, at the same time, preferentially weighting neurons tuned away from θ_h and θ_l. This is evident from the weighting profile w_i provided in Figure S2: the peak and trough of w_i are slightly shifted away from θ_h and θ_l. This a direct result of subtracting two Gaussian-shaped functions, peaking at θ_h and θ_l and with a bandwidth that is not infinitely small. If θ_h and θ_l are far-apart, neurons tuned exactly to θ_h and θ_l have the highest signal-to-noise ratio and the peak and trough of w_i equal θ_h and θ_l. Off-looking thus arises naturally in the optimal log likelihood ratio decoder.

In contrast, the perceptual bias in the present study results from a suboptimal decoding strategy in which considerable weight is assigned to irrelevant neurons, i.e., neurons tuned away from the relevant

grating frequencies. Such a strategy may be called off-looking, too, but the amount of off-looking here is much larger than in case of optimal decoding. As one can see in Figure S2, in the presence of optimal off-looking, most neurons tuned to irrelevant low and high spatial frequencies are ignored. In particular, the optimal log likelihood ratio decoder does not predict a bias (see also section S1.3.2).

1.2.4 Constrained optimality and neural plausibility

The performance of the likelihood ratio decoder is compromised by approximations in the evaluation of the likelihood. As mentioned in section S1.1, we consider a simplified likelihood computation that is ignorant about the filtered noise. More specifically, low and high spatial frequency components in low-pass and high-pass filtered noise are assumed to increase the likelihood of low and high spatial frequencies. A more complex decoder, computing likelihoods via equation 2, can obtain the likelihood function of the *grating* spatial frequency *independent of the noise spatial frequency components.* Such a decoder will not judge the likelihood of a grating spatial frequency to be higher due to the presence of noise. Furthermore, the decoder assumes independent Poisson variability (response variance equal to the mean) although overdispersed Poisson variability (response variance equal to 1.5 times the mean) is assumed in the encoding model.

A likelihood ratio decoder making these simplifying assumptions regarding the statistics of the population code has a major advantage: it can be implemented through linear weighted summation. More specifically, evaluating the (reduced) log likelihood of a specific grating spatial frequency simplifies to summing the responses of neurons tuned to this frequency. To obtain the weighting profiles specified by equations 16-17, the decoder only requires knowledge of which neurons respond strongly to the relevant grating spatial frequencies. Likelihood is encoded implicitly in the responses of these neurons.

Interestingly, even our simplified decoder considerably outperforms human observers by failing to predict a perceptual bias. Every more complex decoder, more compatible with the assumptions of the encoding model, can only outperform the simplified decoder and will therefore fail to account for our results. For instance, the fact that we observe a perceptual bias strongly suggests that the likelihood representation is biased by filtered noise and hence, that filtered noise is not taken into account when computing likelihoods.

The optimal weighting profiles are independent of noise condition but do depend on grating spatial frequency. In the main discrimination experiment, standard and comparison spatial frequencies were therefore kept constant within each block and signal templates are provided at the beginning of each block. The population responses to the templates and to the gratings on each trial can in principle be used to compute the optimal weighting profile. Furthermore, to control for a possible inability to quickly obtain the optimal weighting profile at the beginning of each block, we conducted the control experiment in which only two grating spatial frequencies were included and the same weighting profile can be used throughout the experiment.

1.3 Suboptimal log likelihood ratio decoding

1.3.1 Imprecise likelihood read-out

Optimal log likelihood ratio decoding entails selectively evaluating the likelihood of the grating spatial frequencies that are a-priori likely to occur during the task. Other spatial frequencies should be ignored. Formally, the continuous likelihood function has to be read out at one or two discrete locations. This means that a detailed representation of the relevant discrete spatial frequencies is required as well as knowledge of the subpopulation of neurons tuned to these frequencies (see section S1.2.2). It is not unthinkable that observers are to some extent uncertain about which grating spatial frequencies or neural responses are relevant to the task at hand. We therefore consider a likelihood ratio decoding scheme in which the precision of likelihood function read-out is controlled by a free parameter. This decoding is

similar to the decoder defined in section S1.2 except that the approximate log likelihoods $\log L_s$ are provided by:

$$\log L_s(\theta_0) = \int_{-\infty}^{+\infty} p(\theta) \log L(\theta) d\theta \qquad (20)$$

where θ_0 is a specific spatial frequency and $p(\theta)$ is a Gaussian-shaped weighting function (referred to as read-out function) centred at θ_0 with a bandwidth controlled by a free parameter. Instead of reading out the likelihood function at one exact discrete location, the decoder evaluates the average likelihood over a range of (irrelevant) spatial frequencies specified by $p(\theta)$. In the limit if the width of $p(\theta)$ goes to zero, the imprecision vanishes and the log likelihood ratio decoder becomes optimal.

1.3.2 Low-precision pooling and excessive off-looking

The suboptimal log likelihood ratio decoder can be implemented through linear weighted summation as described in section S1.2.2. Because $p(\theta)$ is Gaussian, the weighting profiles w_i can again be approximated by either a bell-shaped or a difference of bell-shaped functions. However, depending on the width of $p(\theta)$, the weighting profiles used by the suboptimal likelihood ratio decoder may be considerably broader than the optimal weighting profiles. If $p(\theta)$ is broad, evaluating the likelihood of a discrete spatial frequency θ_0 following equation 20 corresponds to summing the responses of a broad population of neurons, including a considerable amount of neurons insensitive to θ_0. This low-precision pooling is illustrated in Figure S3. Note the presence of off-looking when the likelihood function is read out with limited precision, even though the grating spatial frequencies are relatively far apart (Figure S3, blue lines). The reason is that the weighting profile consists of a difference of two broad Gaussian-like functions. Subtracting broader functions results in increased off-looking. This amount of off-looking is excessive: the optimal log likelihood ratio decoder (Figure S3, green lines) does not display off-looking when discriminating between such relatively far-apart spatial frequencies.

1.4 Bayesian inference and the effect of loss functions

The Bayesian framework allows us to decompose optimal decoding into two steps: (1) computing the posterior distribution, and (2) minimizing the expected loss for the given posterior. While we do not know the loss functions that are used by the human observers it is important to note that an imprecise computation of the posterior distributions is critical to explain the perceptual bias. Conversely, if the posterior is computed correctly it is impossible to obtain the bias just by the choice of a particular loss function.

The posterior probability of a specific grating spatial frequency θ having led to the response n_i of neuron i is obtained by multiplying the likelihood of the response given that spatial frequency with the prior probability of the spatial frequency occurring during the perceptual task at hand:

$$p(\theta|n_i) = \frac{1}{\alpha} p(n_i|\theta) p(\theta) \qquad (21)$$

$$= \frac{1}{\alpha} l_i(\theta) p(\theta) \qquad (22)$$

where α is a normalization constant independent of θ that can be safely ignored and $l_i(\theta)$ is defined by equation 3. In the 2-AFC spatial frequency discrimination task, two known grating spatial frequencies θ_h and θ_l occur with equal probability. Therefore, the prior probability function consists of two delta functions centred at θ_h and θ_l:

$$p(\theta) = \begin{cases} 0.5 \text{ if } \theta = \theta_h \text{ or } \theta = \theta_l \\ 0 \text{ otherwise} \end{cases} \qquad (23)$$

The posterior for the population response is given by:

$$p(\theta|\vec{n}) = \prod_{i=1} p(\theta|n_i) \tag{24}$$

where $\vec{n}$ is the population response vector. As it is clear from the definition of the posterior, it is completely independent of the likelihood of all stimuli but exactly the two that are to be discriminated. Therefore, the bias cannot be obtained by the subsequent choice of a particular loss function. Rather it is crucial that the computation of the posterior involves imprecisions that lead to excessive off-looking as explained above.
