# Supplementary material for: A New Perceptual Bias Reveals Suboptimal Population Decoding of Sensory Responses
Source: PLoS Comput Biol. 2012 Apr 12;8(4):e1002453. doi: 10.1371/journal.pcbi.1002453 (PMC3325184; doi:10.1371/journal.pcbi.1002453)
Supplement: Text S2 — Supporting text discussing inter-individual differences and data pooling. (PDF) [file pcbi.1002453.s010.pdf]

Supporting text S2: inter-individual differences and data pooling

Individual data for the discrimination task are provided in Figure S4. Some inter-individual differences were observed. In the main conditions, subject TP and BM tend to display a slightly stronger bias in the low-pass noise condition, while this pattern is reversed for subject KT. In the control conditions, the size of the bias is comparable in low-pass and high-pass noise conditions but is somewhat larger for subject BM compared to subject TP and KT.

As the perceptual bias is significant for all observers and differences are inconsistent across observers, we decided to pool the data using the following procedure [1]. For each observer and experimental condition, we determined the relative matching frequency. The individual matching frequencies were averaged across observers to obtain average matching frequencies for both low-pass and high-pass noise conditions in both main and control conditions. Individual and average matching frequencies were then used to normalize the individual comparison frequency scales by dividing comparison spatial frequencies by the individual matching frequencies and then multiplying by the average matching frequency.

Individual data for the detection task are provided in Figure S5. As inter-individual differences were again small, we pooled the detection data following the same procedure used on the discrimination data.

References

1. Goris RLT, Wagemans J, Wichmann FA (2008) Modelling contrast discrimination data suggest both the pedestal effect and stochastic resonance to be caused by the same mechanism. J Vis 8: 17.1–1721.
