# Supplementary material for: A New Perceptual Bias Reveals Suboptimal Population Decoding of Sensory Responses
Source: PLoS Comput Biol. 2012 Apr 12;8(4):e1002453. doi: 10.1371/journal.pcbi.1002453 (PMC3325184; doi:10.1371/journal.pcbi.1002453)
Supplement: Text S3 — Supporting text discussing contrast sensitivity across spatial frequency. (PDF) [file pcbi.1002453.s011.pdf]

Supporting text S3: contrast sensitivity across spatial frequency

We determined the contrast corresponding to the 84%-correct detection threshold in broadband noise at different grating spatial frequencies for each observer participating in the discrimination experiments. A procedure similar to the procedure used in the main contrast detection experiment (see methods) was used, except that two interleaved one-up-three-down staircases were used to determine the contrast level on each trial. Each staircase consisted of 50 trials. Each observer completed a total of 900 trials. The average contrast of the last 25 trials was used as the threshold contrast estimate. A second-degree polynomial contrast sensitivity function was fitted to relate threshold contrasts to grating spatial frequency separately for each observer (Figure S6). These functions were used to determine the grating contrast levels in the discrimination experiments.

The detection threshold contrasts seem to increase somewhat at higher grating spatial frequencies. As this increase is limited, we did not take it into account in the model for computational convenience. Discrimination performance was simulated at a grating contrast equal to the average of the contrasts used in the discrimination tasks (Figure S6, broken line). This contrast was close to the actual contrasts used. In addition, we normalized the gain of the linear filters implemented in the model to obtain equal sensitivity across preferred spatial frequencies (see methods). Incorporating a more complex contrast sensitivity function would allow the model to capture an asymmetry in the perceptual bias. However, the fit of our ideal observer model, which either underestimates the size of the bias in both low- and high-pass noise conditions or underestimates grating detectability at 5.5 c/deg in the notched noise condition will not benefit from the inclusion of a contrast sensitivity function tilted around 5.5 c/deg.
