# Supplementary material for: A New Perceptual Bias Reveals Suboptimal Population Decoding of Sensory Responses
Source: PLoS Comput Biol. 2012 Apr 12;8(4):e1002453. doi: 10.1371/journal.pcbi.1002453 (PMC3325184; doi:10.1371/journal.pcbi.1002453)
Supplement: Text S4 — Supporting text discussing the role of across-trial neural response variability. (PDF) [file pcbi.1002453.s012.pdf]

Supplementary text S4: the role of across-trial variability

Various studies have used a Poisson noise model to describe neural firing statistics. In this model, response variance equals response mean and external noise only indirectly affects response variability through changes in mean response rate. As the main effect of external noise seems to be an inhibitory decrease of the mean response (see main text), external noise is expected to reduce response variability. However, individual external noise backgrounds vary considerably across trials and are likely to introduce variability in mean response rate. Recent evidence [1] suggests that such across-trial variability can lead to a increase in the ratio of response variance to mean, also referred to as the Fano factor. To avoid underestimation of the total amount of response variance, we used a Fano factor of 1.5 in our simulations. This value is relatively large compared to a value of 1 (which is expected in the absence of across-trial variability) but not uncommon [2].

Importantly, we found that changing the exact value of the Fano factor does not change the conclusions of our study. Figure S7 shows predicted discrimination performance for the constrained model (left panel) and unconstrained model (right panel) when a Fano factor of 1 (dotted lines) instead of 1.5 (full lines) is assumed while other parameters are kept at the best-fitting values. Predicted detection performance is provided in Figure S8. The considerable change in Fano factor clearly has little impact on model predictions. We also refitted the constrained model assuming a Fano factor of 1 in the encoding stage. Goodness-of-fit of this model remains significantly lower compared to the unconstrained model ($\chi^2 = 153.75$, parametric Monte-Carlo test, $p < 0.01$). These results suggest that the behavioural effects of external noise in our experiments are mainly due to noise-induced changes in mean response instead of an increase in across-trial variability.

References

1. Churchland AK, Kiani R, Chaudhuri R, Wang XJ, Pouget A, et al. (2011) Variance as a signature of neural computations during decision making. Neuron 69: 818–831.

2. Geisler W, Albrecht D (1997) Visual cortex neurons in monkeys and cats: detection, discrimination and identification. Vis Neurosci 14: 897–920.
